# CD248 deficiency promotes angiotensin II‐induced aortic lesion by attenuating receptor stability in smooth muscle cells

**DOI:** 10.1002/ctm2.70352

**Published:** 2025-05-25

**Authors:** Tai‐Tzu Hsieh, Ya‐Chu Ku, Chu‐Jen Chen, Cheng‐Hsiang Kuo, Bi‐Ing Chang, Chien‐Hung Yu, Yi‐Heng Li, Pei‐Jane Tsai, Shu‐Wha Lin, Hua‐Lin Wu, Chwan‐Yau Luo, Yau‐Sheng Tsai

**Affiliations:** ^1^ Institute of Clinical Medicine College of Medicine National Cheng Kung University Tainan Taiwan, ROC; ^2^ Institute of Basic Medical Sciences College of Medicine National Cheng Kung University Tainan Taiwan, ROC; ^3^ Department of Biochemistry and Molecular Biology College of Medicine National Cheng Kung University Tainan Taiwan, ROC; ^4^ Department of Internal Medicine National Cheng Kung University Hospital, College of Medicine National Cheng Kung University Tainan Taiwan, ROC; ^5^ Department of Medical Laboratory Science and Biotechnology College of Medicine National Cheng Kung University Tainan Taiwan, ROC; ^6^ Department of Clinical Laboratory Sciences and Medical Biotechnology College of Medicine National Taiwan University Taipei Taiwan, ROC; ^7^ Division of Cardiovascular Surgery Department of Surgery Kaohsiung Medical University Hospital Kaohsiung Taiwan, ROC; ^8^ Department of Surgery College of Medicine, Kaohsiung Medical University Kaohsiung Taiwan, ROC; ^9^ Clinical Medicine Research Center National Cheng Kung University Hospital Tainan Taiwan, ROC

**Keywords:** aortic aneurysm, vascular smooth muscle cells, collagen, angiotensin II receptors, PDGF receptors, CD248

## Abstract

**Background:**

Abdominal aortic aneurysm (AAA) is characterized by progressive dilation of the abdominal aorta that has a high prevalence of death due to aortic rupture. The hallmark of AAA is severe degeneration of the aortic media with the loss of vascular smooth muscle cells (VSMCs), the main source of extracellular matrix (ECM) proteins. CD248 was originally implicated in angiogenesis and tumourigenesis, but its role in the development of AAA remains unclear.

**Methods:**

Mice lacking CD248 (*Cd248* ^−/−^) were generated and evaluated for angiotensin II (Ang II) and high‐cholesterol diet feeding induced AAA. Loss‐of‐function approaches in A7r5 and C3H10T1/2 cells were used to study the involvement of CD248 in the Ang II signalling.

**Results:**

CD248 expression was upregulated in the media and adventitia of patients and mice with aortic aneurysm. CD248 deficiency in mice exacerbates Ang II‐induced aortic lesion along with severe disruption of elastic fibres and the VSMC layer. Interestingly, while compensatory ECM deposition was found in the aortic lesion of *Cd248* ^−/−^ mice, collagen I content and p38 activation were significantly attenuated. Silencing of CD248 in VSMCs downregulated mitogen‐activated protein kinase activation and ECM production. Loss of CD248 in VSMCs destabilized the membrane receptors for Ang II and platelet‐derived growth factor (PDGF), and the C‐terminal cytoplasmic domain of CD248 is apparently involved in this interaction.

**Conclusions:**

The findings reveal that CD248 regulates the stability of the membrane receptors for Ang II and PDGF in VSMCs to transduce signals for collagen production in combating the loss of aortic wall strength during vascular remodelling.

**Key points:**

- CD248 reduces the occurrence of angiotensin II (Ang II)‐induced aortic lesion by facilitating collagen production to provide load‐bearing properties to the aortic wall.
- CD248 regulates the stability of the membrane receptors for Ang II and PDGF in VSMCs.
- The C‐terminal cytoplasmic tail of CD248 is a crucial domain that potentially regulates the stability of Ang II and PDGF receptors.
- This knowledge can enhance our understanding of how abdominal aortic aneurysm (AAA) can be treated through CD248‐mediated signaling to maintain aortic wall strength during the remodeling process.

## Graphical abstract

1. In the established angiotensin II‐induced AAA model, CD248 deficiency exacerbated aortic lesion, accompanied by lower collagen I content and p38 activation.

2. Silencing CD248 in VSMCs led to reduced MAP kinase activation and ECM production.

3. Furthermore, loss of CD248 in VSMCs destabilizes membrane receptors for angiotensin II and PDGF, a phenomenon possibly mediated by its C‐terminal cytoplasmic tail.

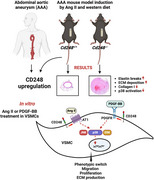

## 1 INTRODUCTION

Abdominal aortic aneurysm (AAA) is a common disease that is characterized by progressive dilation of the abdominal aorta. As most AAAs do not produce clear symptoms, there is a high prevalence of sudden aortic rupture, with a significantly high mortality rate of 80%.^1^, ^2^ An aneurysm is defined as a permanent localized dilation of the aorta that is thought to be caused by adverse remodelling of the aortic wall. This remodelling involves an initial loss of structural integrity as a result of vascular smooth muscle cell (VSMC) apoptosis and a reduction in elastic laminae, followed by dilation of the fibrotic adventitia of the aortic wall.^3^, ^4^ Compensatory deposition of collagen is a hallmark of aortic aneurysm, and defective extracellular matrix (ECM) deposition is believed to be associated with AAA rupture.^5^, ^6^

The most prominent feature of AAA is degeneration of the medial layer due to elastin fibre degradation and loss of VSMCs.^7^ VSMCs are considered contractile to maintain vascular tone in a healthy arterial wall, whereas, in pathological conditions such as AAA, they dedifferentiate into a synthetic phenotype to secrete large quantities of ECM proteins, proliferate and migrate to repair injury.^7^ During AAA pathogenesis, the loss of VSMCs also retards the intrinsic repair mechanism, because VSMCs are the major cell type responsible for the biosynthesis of elastin and other ECM proteins in the vessel wall. Adventitial fibroblasts and myofibroblasts also synthesize components for fibrillar collagen, which provides structural integrity and mechanical strength of the vessel wall.^8^ Thus, fibroblasts also contribute to the synthesis of ECM proteins, particularly in the AAA, whereby VSMCs are downregulated.

CD248, also called endosialin or tumour endothelial marker 1, is a highly glycosylated type I transmembrane protein belonging to the Group XIV C‐Type lectin family. Although CD248 was previously identified as a marker of tumour endothelial cells, it is highly expressed in perivascular mural cells, such as pericytes, VSMCs, and fibroblasts. However, its expression is rather low at the basal level. Stromal CD248 mediates microvascular maturation during tumourigenesis and promotes fibroblast proliferation during fibrogenesis.^9^, ^10^ Furthermore, CD248 interacts with the ECM and cytoskeleton, and regulates signal transduction and cellular behaviors.^10^, ^11^ In atherosclerosis, CD248 deficiency shifts the balance towards a contractile VSMC phenotype and attenuates plaque development.^12^ Importantly, CD248 has been implicated in the platelet‐derived growth factor (PDGF) signalling cascade in regulating pericyte and hepatic stellate cell proliferation. Additionally, CD248‐expressing myofibroblasts, in combination with PDGFRα, enhance PDGF‐BB‐induced mitogenic and chemoattractive effects and promote collagen deposition in wound healing.^13^, ^14^, ^15^ Therefore, the role of CD248 in disease progression is complex, partly due to its pleiotropic function as a co‐receptor for certain receptors.

Because one of the histopathological hallmarks of AAA is severe degeneration of the elastic media with extensive loss of VSMCs, this results in a decreased ability of VSMC to produce ECM to maintain the strength of the aortic wall. Therefore, we hypothesized that CD248 participates in the progression of aortic aneurysms due to its pleiotropic nature in coordinating several membrane receptors. In this study, we demonstrated that the loss of CD248 promoted angiotensin II (Ang II)‐induced aortic lesion and attenuated elastin and collagen fibre component production in mice. In vitro, we found that CD248 stabilizes certain membrane receptors and enhances downstream signalling for ECM production in VSMCs. These results may help unveil the pleiotropic nature of CD248 in VSMC to maintain aortic wall strength.

## 2 METHODS

### 2.1 Human subjects and aortic tissue

Human aortic samples were collected during open aneurysmectomy. Samples at least 6 cm in diameter from eight patients with aortic aneurysm, including three AAA, three thoracic aortic aneurysms, and two root aneurysms, were used for immunohistochemical staining. Sections of control human aorta were taken from aortic valve surgery (aortic valve three leaflets). Samples were obtained from three patients with severe aortic stenosis without aortic dilation, and verified by the pathologist with non‐tumour structures and within normal limits. All participants provided written informed consent for this study, which was approved by the Institutional Review Board (B‐ER‐108‐284) of National Cheng Kung University Hospital and conformed to the principles of the Declaration of Helsinki. The patient information, including age, sex, surgical site of the aneurysms and the presence of intraluminal thrombus, was de‐identified and is included in Tables S1 and S2.

### 2.2 Animal models and treatments

We generated *Cd248*‐deficient (*Cd248* ^−/−^) mice, in which the *LacZ* gene was targeted to the *Cd248* locus to replace *Cd248* and maintained on a C57BL/6JNarl background (obtained from National Laboratory Animal Center, Taiwan).^16^ Six to seven‐month‐old male *Cd248* ^+/+^ and *Cd248* ^−/−^ littermates were used in all experiments. The following groups were studied: (1) No Ang II infusion in *Cd248* ^+/+^ and *Cd248* ^−/−^ mice, (2) Ang II (1000 ng/kg/min, A9525, Sigma‐Aldrich) infusion^1^, ^17^ and high‐cholesterol diet (1.5 g cholesterol, D12079Bi, Research Diets) feeding in *Cd248* ^+/+^ and *Cd248* ^−/−^ mice. Mice at 5–6 months of age were anaesthetized and subcutaneously implanted with ALZET osmotic minipumps (1004; DURECT Corporation), delivering Ang II for 28 days.^1^, ^17^ Mice were euthanized with anaesthetic overdose at 28 days after pump implantation, and aortic tissues were analysed. Mice were anaesthetized by intraperitoneal injection of Zoletil‐Rompun mixture (1 mL Zoletil® (50 mg/mL) +  .1 mL Rompun® + 3.9 mL normal saline), with the dosage of  .1 mL mixture per 20 g mouse body weight. Mice were housed in a specific pathogen‐free barrier facility with the humidity and temperature controlled. All animal studies and anaesthetic and analgesic agents were performed according to protocols approved by the Institutional Animal Care and Use Committee of National Cheng Kung University (107155 and 110203).

### 2.3 Data analysis

Values are reported as mean ± SEM. Statistical analyses were conducted by Student's *t*‐test for parametric data and Mann–Whitney *U* test for non‐parametric data, and one‐way ANOVA or two‐way ANOVA with time and genotype as factors followed by Bonferroni correction. The analyses of aortic lesion incidence and severity grading are conducted by Pearson's chi‐squared (χ^2^) test. Statistical significance was set at *p* value < .05.

A detailed description of the methods and materials are provided in the Supporting Information.

## 3 RESULTS

### 3.1 CD248 upregulation in the aorta of humans and mice

**FIGURE 1:**
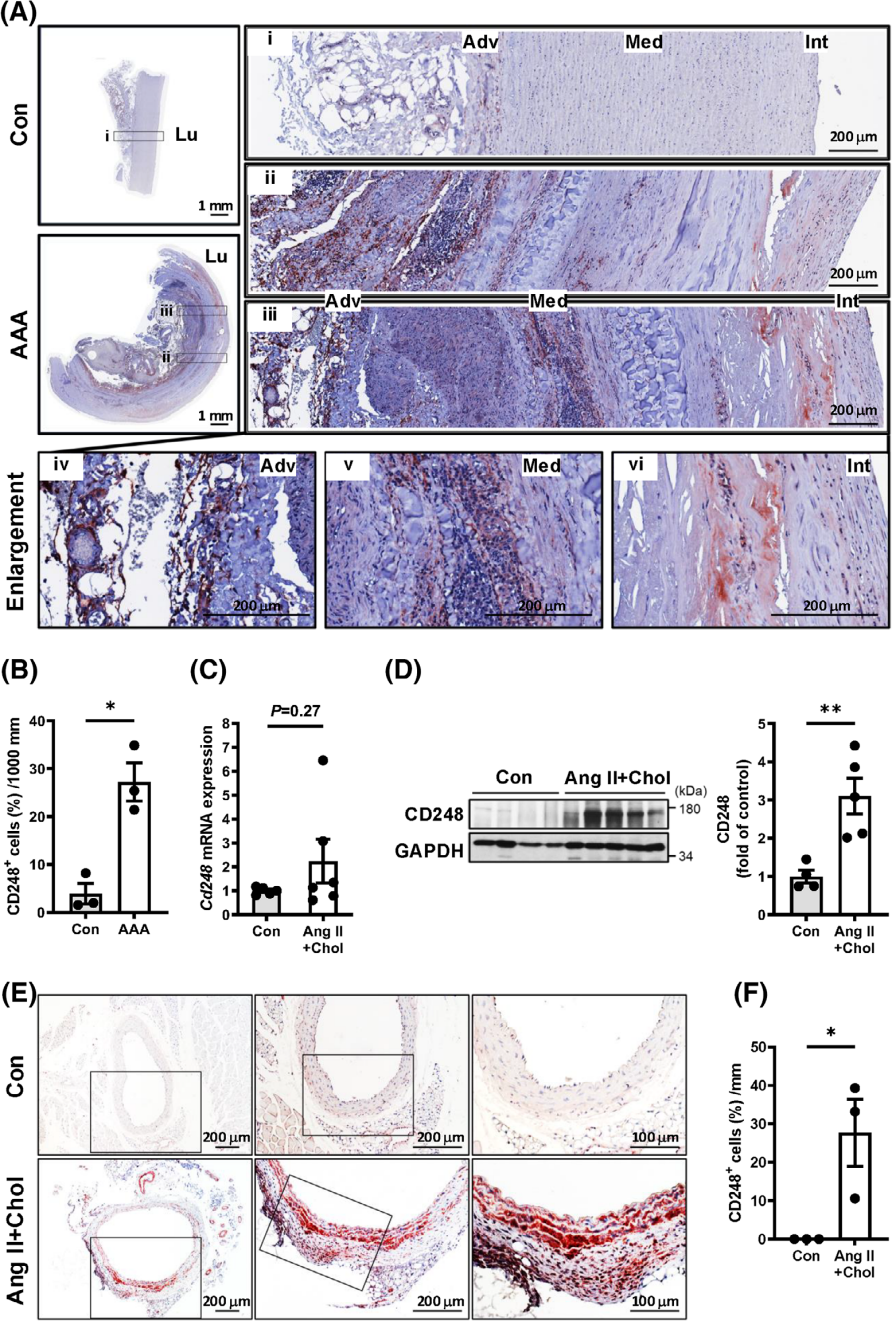
Upregulation of CD248 in human and mouse abdominal aortic aneurysm (AAA). (A) Representative immunohistochemical staining for CD248 in control human abdominal aortic tissues and AAAs. The magnified versions of the images in the black squares in the left panels are shown in the respective right panels (i–iii). The magnified versions of the iii images are shown in the respective lower panels (iv–vi). (B) Quantification of CD248‐positive areas in human controls and aneurysm aortas (*N* = 3). (C) mRNA levels of CD248 in Ang II+Chol *Cd248* ^+/+^ aorta are shown relative to the mean level in control *Cd248* ^+/+^ aorta (*N* = 5–6). (D) Immunoblotting and quantification of CD248 in the abdominal aorta (AA) of control mice (Con; *N* = 4 male) and Ang II‐infused mice fed a high‐cholesterol diet (Ang II+Chol; *N* = 5 male). (E) Representative immunohistochemical staining and quantification (F) for CD248 in the AA of control and Ang II+Chol mice (*N* = 3). The magnified versions of the images in the black squares in the left panels are shown in the respective right panel. Scale bars of 1 mm, 200 µm, and 200 µm are indicated, respectively. *p < .05 and **p < .01 by Mann–Whitney *U* test in (B), (C), (D), and (F). Adv, adventitia; Int, intima; Lu, lumen; Med, media.

To explore whether CD248 is involved in the pathophysiology of aortic aneurysms, we first investigated its expression in patients with aortic aneurysm and compared Ang II (1000 ng/kg/min)‐infused mice fed a high‐cholesterol diet (Ang II+Chol) against untreated conrtol mice. In humans, immunohistochemical staining revealed that normal human aortic samples scarcely express CD248. In contrast, CD248 expression was significantly increased in the medial smooth muscle and adventitial fibroblast layers of patients with aortic aneurysm, despite minimal VSMCs in advanced stage aneurysmal tissues (Figure 1A,B; Figures S1 and S2). In the murine model, we focused on the expression and location of CD248 protein in abdominal aortic tissues from Ang II+Chol mice. Although Ang II+Chol treatment in mice for 4 weeks did not significantly increase *Cd248* mRNA level (Figure 1C), the core protein of mouse CD248 (approximately 95 kDa), as well as its form with post‐translational modifications (approximately 165 kDa), was upregulated after 28 days of Ang II+Chol treatment (Figure 1D). Consistently, CD248 expression was significantly induced in the media and adventitia, especially within the dilated aorta, in response to Ang II+Chol treatment (Figure 1E,F). Thus, upregulated expression of CD248 in human and mouse aortic lesion suggests that CD248 may participate in aortic aneurysm formation and progression.

### 3.2 CD248 deficiency exacerbates Ang II+Chol‐induced aortic lesion

**FIGURE 2:**
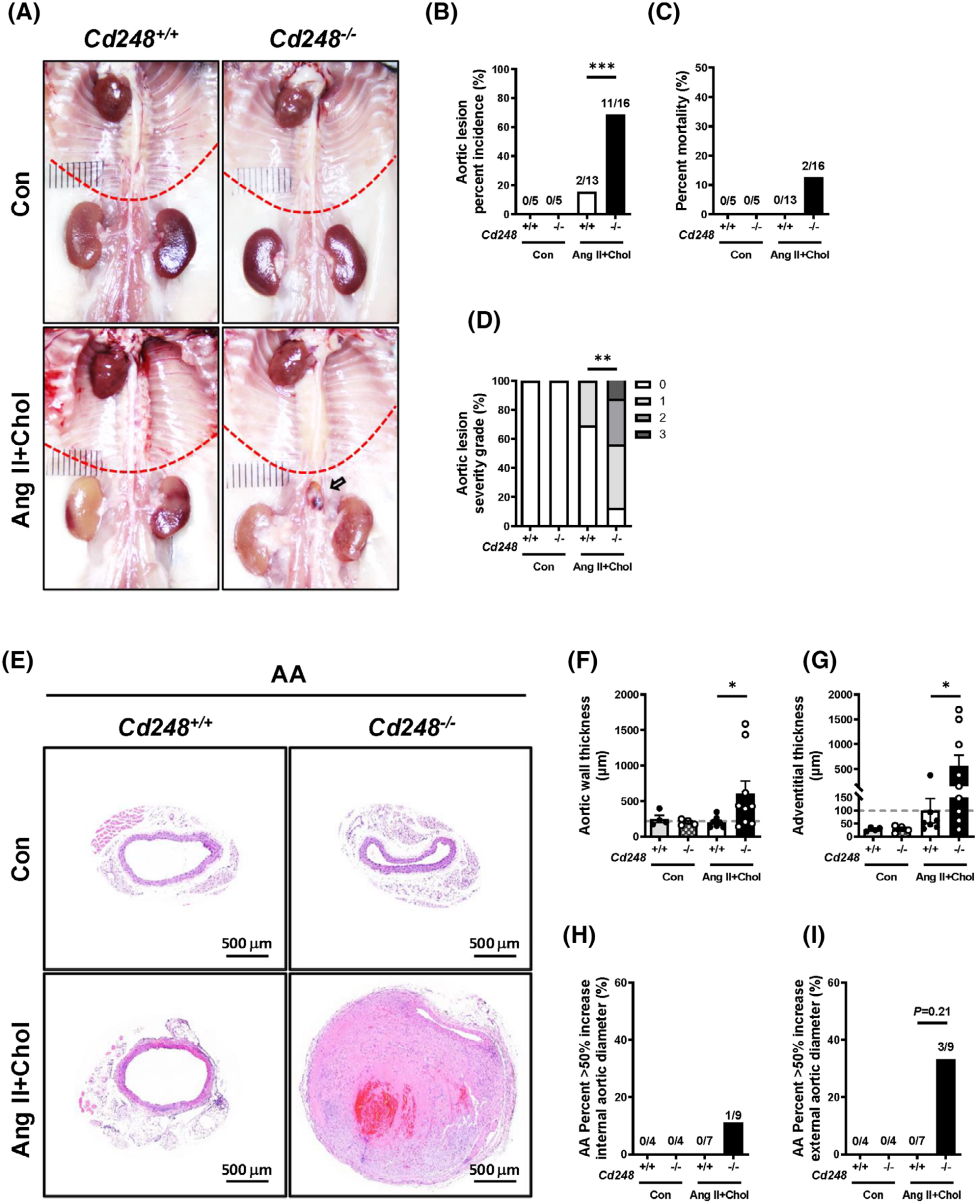
Exacerbated abdominal aortic lesion in *Cd248* ^−/−^ mice. (A) Representative photographs of the aorta. The red dotted line indicates the diaphragm. Arrow indicates abdominal aortic lesion. Incidence rate of abdominal aortic lesion (B). Percentage of mortality (C) and severity grade (D) in Ang II+Chol *Cd248* ^+/+^ and *Cd248* ^−/−^ male mice (Con; *N* = 5, Ang II+Chol; *N* = 13–16). (E) Representative haematoxylin and eosin (H&E) staining of abdominal aorta (AA). (F, G) Aortic wall thickness and adventitial thickness; (H, I) the percentage of AA > 50% max internal and external aortic diameter in Ang II+Chol *Cd248* ^+/+^ and *Cd248* ^−/−^ male mice (Con; *N* = 4, Ang II+Chol; *N* = 7–9). Scale bars are 500 µm in (E). *p < .05, **p < .01, and ***p < .005 by Pearson's chi‐squared (χ^2^) test in (B), (C), (D), (H), and (I) and by Student's *t*‐test in (F) and (G).

To directly test whether CD248 affects aneurysm formation, we generated *Cd248*‐deficient (*Cd248* ^−/−^) mice. Systolic blood pressure (SBP), diastolic blood pressure, mean blood pressure, and heart rate did not differ between the genotypes in the basal state (Figure S3A–D). No aortic lesion was observed in the control *Cd248* ^+/+^ and *Cd248* ^−/−^ mice (Figure 2A,B). Ang II+Chol treatment increased SBP in both genotypes but did not result in differences in SBP, diastolic blood pressure, mean blood pressure, and heart rate between genotypes. While Ang II+Chol treatment induced AAA in *Cd248* ^−/−^ mice, its effect was much less severe in *Cd248* ^+/+^ mice (Figure 2A). Quantification showed that 1 of 13 (7.69%) *Cd248* ^+/+^ mice developed a thoracic aortic lesion, whereas 5 of 16 (31.25%) *Cd248* ^−/−^ mice developed thoracic aortic lesion, but the difference did not reach statistical significance (Figure S4A–L). Moreover, 2 of 13 (13.38%) *Cd248* ^+/+^ mice developed abdominal aortic lesion, and 11 of 16 (68.75%) *Cd248* ^−/−^ mice developed abdominal aortic lesion (Figure 2B). At the 28‐day time point, the mortality rate was increased to 2/16 (12.5%) in *Cd248* ^−/−^ mice compared to 0/13 (0%) in *Cd248* ^+/+^ mice (Figure 2C). We defined the severity grade of thoracic aortic lesion/abdominal aortic lesion by the maximal external diameter^17^ (Figure S4C and Figure 2D) and luminal diameter (Figures S4D and S5A). Histological analyses of the cross‐sections of the thoracic (Figure S4A) and abdominal aorta (Figure 2E) did not identify gross differences between the control *Cd248* ^+/+^ and *Cd248* ^−/−^ aortas. Ang II+Chol treatment induced vascular hypertrophy in the thoracic aorta (Figure S4A–L) and abdominal aorta (Figure 2E) of *Cd248* ^−/−^ mice. With a marked contrast, the aortic wall and adventitial thicknesses of the abdominal aorta were significantly enlarged with a trend towards increase of external aortic diameter in *Cd248* ^−/−^ mice at the 28‐day time point (Figure 2F,G and Figure S5B–E). An AAA is defined as a localized dilatation of the abdominal aorta exceeding the normal diameter by more than 50%.^18^ Ang II+Chol treatment induced abdominal aorta diameters measuring >50% max internal (1/9, 11.1%) and external (3/9, 33.3%) aortic diameter in *Cd248* ^−/−^ mice versus 0% (0/7) in *Cd248* ^+/+^ mice (Figure 2H,I). These differences were not accompanied by changes in plasma triglyceride, free fatty acid, total cholesterol, glucose levels or body weight (Figure S6A–E) between Ang II+Chol‐treated *Cd248* ^+/+^ and *Cd248* ^−/−^ mice. In addition, we also performed Ang II treatment alone and cholesterol feeding alone groups, and found that Ang II treatment alone, but not cholesterol feeding, was sufficient to induce aortic lesion in *Cd248* ^−/−^ mice (Figure S7). Consistent with the previous study showing the relative resistance to Ang II‐induced AAA in female mice,^19^ neither *Cd248* ^+/+^ nor *Cd248* ^−/−^ female mice developed Ang II‐induced aortic lesion (Figure S8). These results suggest that *Cd248* deficiency exaggerates thoracic and abdominal aortic lesion in response to Ang II stimulation.

### 3.3 CD248 deficiency attenuates elastin and collagen fibre components in the aorta after Ang II+Chol induction

**FIGURE 3:**
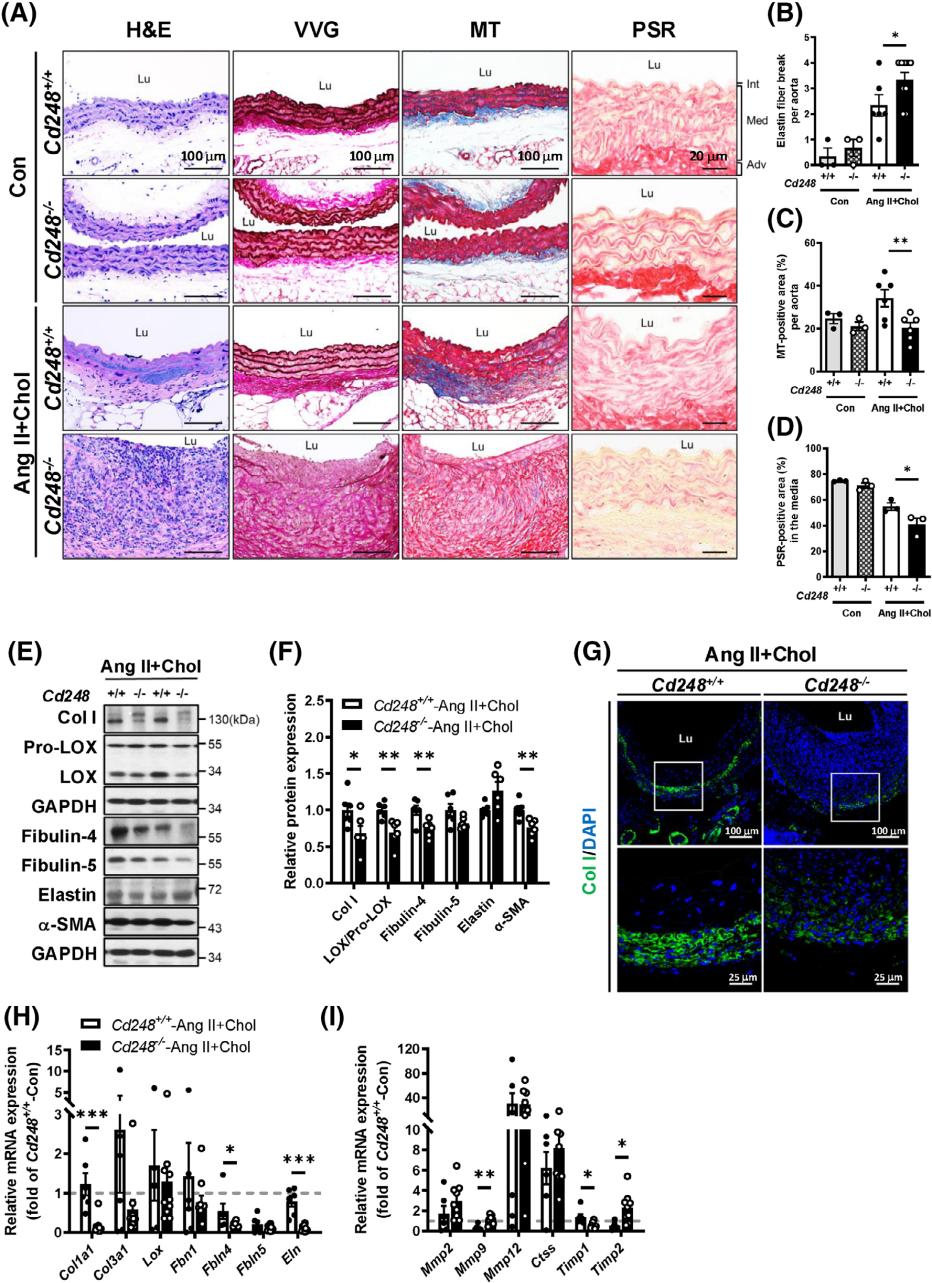
Increased elastic fibre breakage but attenuated collagen deposition in *Cd248* ^−/−^ aorta in response to Ang II+Chol. (A) Histological analysis of abdominal aorta (AA). Left first panels: Representative photographs of the AA cross‐sections with H&E staining to illustrate the extent of abdominal aortic lesion progression. Left second panels: Representative images of Verhoeff‐Van Gieson (VVG) staining demonstrate the extent of elastin fibre break. Left third panels: Representative images of Masson's trichome (MT) staining illustrate the extent of collagen deposition (*blue*). Left fourth panels: Representative images of Picrosirius red (PSR) staining illustrate the extent of collagen fibres (*red*). (B) Quantitation of elastin fibre breakage by VVG staining (Con; *N* = 3, Ang II+Chol; *N* = 6–9). (C) Quantitation of collagen‐positive area of aortic sections by MT staining (Con; *N* = 3, Ang II+Chol; *N* = 6). (D) Quantitation of collagen‐positive area in the media of aortic sections by PSR staining (Con; *N* = 3, Ang II+Chol; *N* = 3). (E) Representative immunoblot and quantification (F) of elastin and collagen fibre components and α‐SMA in the AA (*N* = 6). The relative intensities of the bands are indicated by densitometric quantification with Ang II+Chol *Cd248* ^+/+^. (G) Immunofluorescence staining of Col I (*green*) and DAPI (*blue*) in the AA. (H) mRNA levels of collagens and elastic fibres components and elastolytic enzymes (I) in Ang II+Chol *Cd248* ^−/−^ and *Cd248* ^+/+^ aorta are shown relative to the mean level in control *Cd248* ^+/+^ aorta, which is set as 1.0 (Con; *N* = 4–5, Ang II+Chol; *N* = 6–9). Scale bars of 100, 25, and 20 µm are indicated, respectively. *p < .05, **p < .01, and ***p < .001 by Student's *t*‐test in (B), (C), (F), (H), and (I) and by Mann–Whitney *U* test in (D). Lu: lumen.

Abdominal aortic lesions were analysed for general appearance by haematoxylin and eosin (H&E), elastin lamellae by Verhoeff‐Van Gieson (VVG), and collagen deposition by Masson's trichrome (MT) staining. No visible differences in the basal state were observed between *Cd248* ^−/−^ and *Cd248* ^+/+^ mice (Figure 3A). While Ang II+Chol treatment severely decreased smooth muscle cell density (Figure 3A, H&E staining) and destroyed the elastic lamellae in the medial layer of *Cd248* ^−/−^ mice (Figure 3A and Figure S9A, VVG staining; and Figure 3B and Figure S9C), it modestly affected these in *Cd248* ^+/+^ mice. In addition, the compensatory deposition of collagen (Figure 3A and Figure S9B, MT staining; and Figure 3C and Figure S9D) and infiltration of immune cells (Figure 3A, H&E staining; and Figure S10) occurred modestly in the adventitia of Ang II+Chol‐treated *Cd248* ^+/+^ mice. Massive ECM deposition and immune cell infiltration were the hallmarks of the hypertrophic adventitia observed in *Cd248* ^−/−^ mice. Surprisingly, collagen (*blue*) deposition was observed in Ang II+Chol‐treated *Cd248* ^+/+^ mice but not in *Cd248* ^−/−^ mice (Figure 3C and Figure S9D). In addition, we applied Picrosirius red staining and confirmed that the collagen signal was attenuated in the media of *Cd248* ^−/−^ mice following Ang II+Chol treatment (Figure 3A,D). These results indicate that CD248 deficiency accelerates VSMC loss, elastic lamellar degradation, leukocyte recruitment, and ECM accumulation, which are typical features of AAA. However, the lack of compensatory collagen deposition is another hallmark of vascular remodelling in *Cd248* ^−/−^ mice.

Next, we examined elastin and collagen fibre components in Ang II+Chol‐treated *Cd248* ^+/+^ and *Cd248* ^−/−^ aortas. Elastic fibres are formed by the initial synthesis of the soluble precursor elastin and later maturation by cross‐linking fibulin (fibulin‐4 and fibulin‐5) and fibrillin (fibrillin‐1) scaffold with the elastin core. *Cd248* ^−/−^ mice had lower fibulin‐4 and fibulin‐5 protein levels than *Cd248* ^+/+^ mice (Figure 3E,F). Collagen fibres are formed by Col I and Col III and cross‐linked by lysyl oxidase (LOX), which is cleaved from the proenzyme proLOX. We found that *Cd248* ^−/−^ mice had lower protein levels of Col I, LOX, and a contractile SMC‐specific marker, α‐SMA, than *Cd248* ^+/+^ mice. Immunofluorescence staining confirmed the expression of Col I surrounding the aorta in Ang II+Chol‐treated *Cd248* ^+/+^ mice; however, the Col I signal was significantly attenuated in *Cd248* ^−/−^ mice (Figure 3G). Consistently, mRNA levels of the components for elastin and collagen fibres were generally downregulated in Ang II+Chol‐treated *Cd248* ^−/−^ aorta (Figure 3H). Moreover, CD248 deficiency in mice significantly upregulated *Mmp9* and *Timp2*, and tended to upregulate *Mmp2*, whereas it significantly downregulated *Timp1* (Figure 3I). These results demonstrate that CD248 deficiency not only attenuates several elastin and collagen fibre components but also induces a pro‐degradation profile in response to Ang II+Chol.

### 3.4 CD248 is predominantly induced in VSMC in response to Ang II+Chol

**FIGURE 4:**
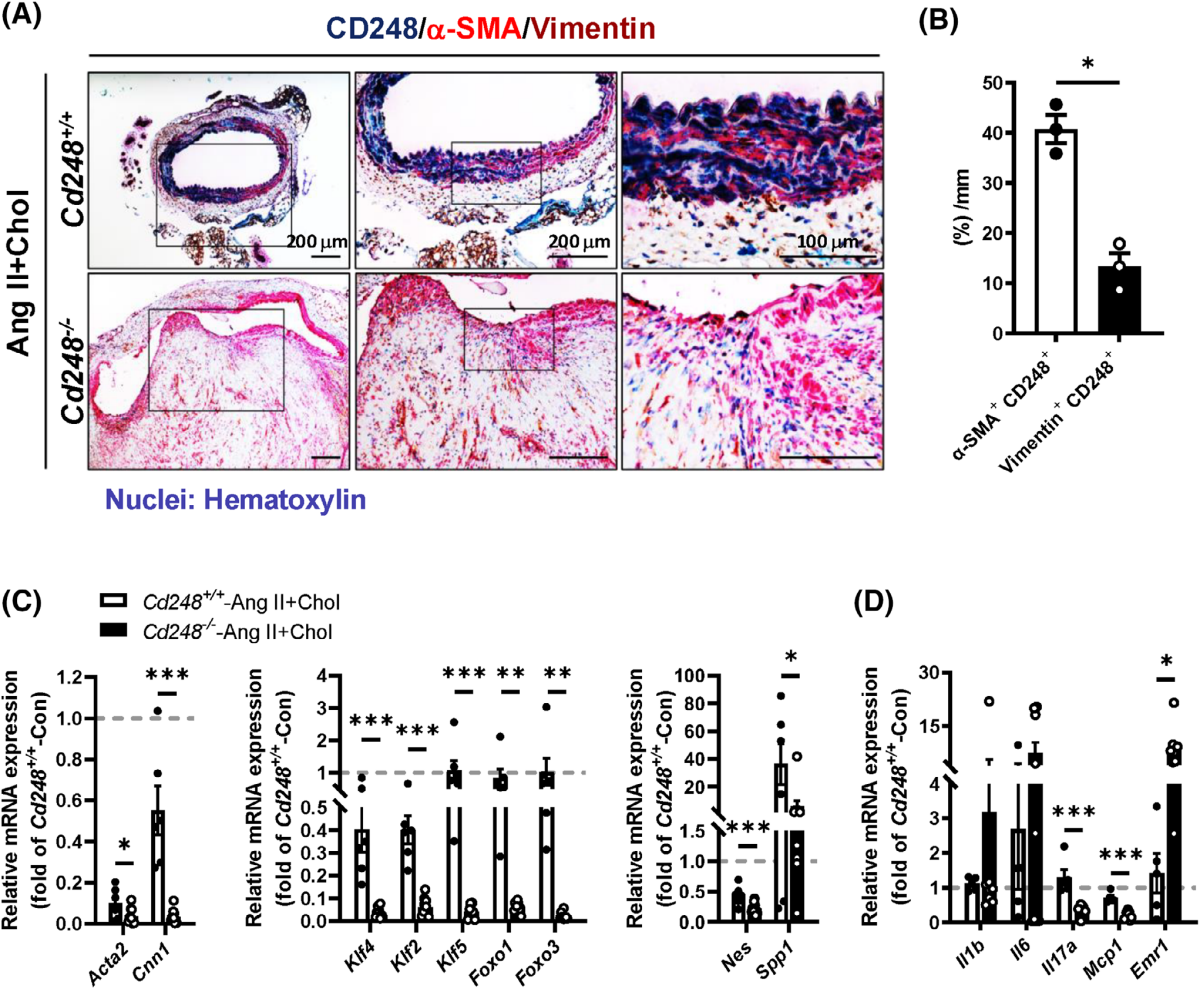
Increased vascular smooth muscle cell (VSMC) loss in *Cd248* ^−/−^ aorta in response to Ang II+Chol. (A) Immunohistochemical staining and quantification (B) for CD248 (*blue*), α‐SMA (*red*), and vimentin (*brown*) of AA in Ang II+Chol *Cd248* ^+/+^ (upper panels) and *Cd248* ^−/−^ (lower panels) mice. The magnified versions of the images in the black squares in the left panels are shown in the respective right panels. *Cd248* ^+/+^ aorta exhibits CD248 (*blue*) expression prominently in α‐SMA (*red*)‐positive cells of the media and modestly in vimentin (*brown*)‐positive cells of the adventitia. The haematoxylin stains cell nuclei *purplish blue*. (C) mRNA levels of VSMC phenotypic markers and transcription factors in Ang II+Chol *Cd248* ^−/−^ and *Cd248* ^+/+^ aorta are shown relative to the mean level in control *Cd248* ^+/+^ aorta, which is set as 1.0 (Con; *N* = 4–5, Ang II+Chol; *N* = 6–9). Contractile VSMC markers: α‐smooth muscle actin (*Acta2*) and calponin (*Cnn1*); and synthetic VSMC marker: nestin (*Nes*) and osteopontin (*Spp1*). (D) mRNA levels of inflammatory cytokines and macrophage markers in Ang II+Chol *Cd248* ^−/−^ and *Cd248* ^+/+^ aorta are shown relative to the mean level in control *Cd248* ^+/+^ aorta, which is set as 1.0 (Con; *N* = 4–5, Ang II+Chol; *N* = 6–9). Macrophage markers: F4/80 (*Emr1*) and CD68. Scale bars are 200 µm in the left four panels and 100 µm in the right two panels. *p < .05, **p < .01, and ***p < .001 by Mann–Whitney *U* test in (B) and by Student's *t*‐test in (C) and (D).

Next, we examined the expression of CD248 in the mouse aorta in response to Ang II+Chol. Immunostaining confirmed a dramatic decrease in the α‐SMA‐positive region (Figure 4A, *red*; VSMCs) and an evident increase in vimentin‐positive area (Figure 4A, *brown*; fibroblasts) in *Cd248* ^−/−^ mice compared to *Cd248* ^+/+^ mice. Moreover, CD248 was predominantly expressed in the α‐SMA‐positive region (40.8%) (Figure 4B and Figure S11A), and modestly expressed in the vimentin (13.3%) and von Willebrand Factor (vWF)‐positive area (Figure 4B and Figure S11B). In addition, F4/80^+^ (*brown*) CD248^+^ (*blue‐green*) double positive cells count for only 12.9% of total F4/80^+^ positive cells (Figure S12). Thus, in response to Ang II+Chol, CD248 was predominantly induced in medial VSMCs and modestly in adventitial fibroblasts, endothelial cells and macrophages. In the aorta from the AAA patient, we confirmed the expression of CD248 in α‐SMA‐positive cells by immunofluorescence staining, and found that CD248‐positive signal was seldom exhibited in the vWF‐positive cells (Figure S13).

Because CD248 is predominantly induced in VSMC, we then examined VSMC phenotypic switching between contractile and synthetic phenotypes. Expression of genes encoding contractile phenotype markers α‐SMA (*Acta2*) and calponin‐1 (*Cnn1*) were markedly downregulated in Ang II+Chol‐treated *Cd248* ^−/−^ aorta (Figure 4C). Notably, transcription factors associated with phenotypic switching, including KLF‐4, KLF‐2, KLF‐5, FOXO‐1, and FOXO‐3, as well as the synthetic phenotype markers nestin (*Nes*) and osteopontin (*Spp1*), were also downregulated in Ang II+Chol‐treated *Cd248* ^−/−^ aorta (Figure 4C). These results suggest that CD248 deficiency not only dampens the pool of contractile VSMCs but also hinders the ability of contractile VSMCs to undergo a phenotypic switch to the osteogenic, ECM‐producing, synthetic state. Immune cell infiltration and proinflammatory cytokine secretion can severely change the VSMC plasticity.^20^ CD248 deficiency increased macrophage marker *Emr1*, and tended to increase *Il1b* and *Il6*, but downregulated *Mcp1* and *Il17a*, in the aorta (Figure 4D).

### 3.5 CD248 deficiency attenuates p38 activation and the levels of PDGFRs and ATs

**FIGURE 5:**
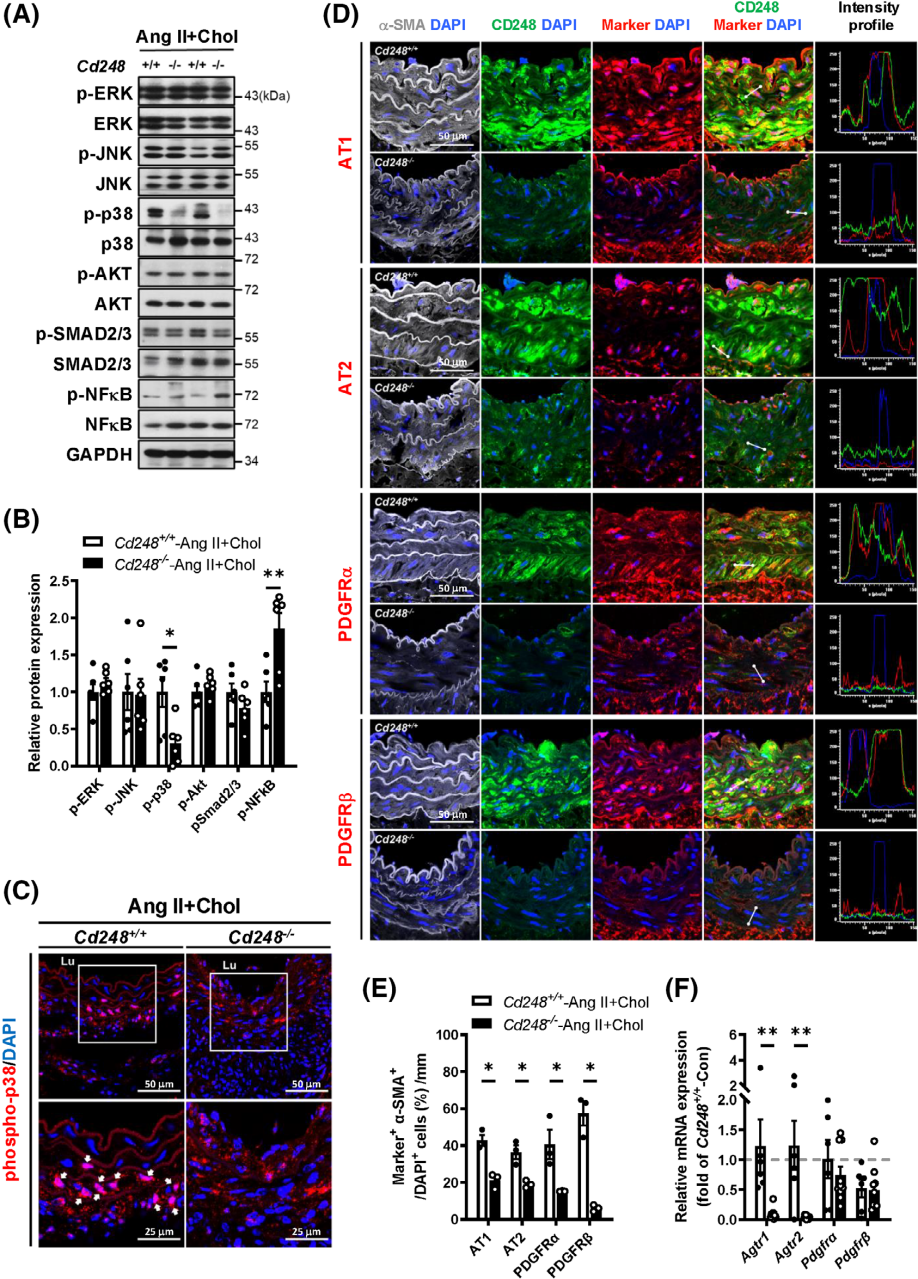
Attenuated p38 MAP kinase activation and the levels of ATs and PDGFRs in *Cd248* ^−/−^ aorta in response to Ang II+Chol. (A) Representative immunoblot and quantification (B) of Ang II downstream signalling molecules in the AA (*N* = 6). (C) Immunofluorescence staining of p‐p38 (*red*) and DAPI (*blue*) in the AA. The magnified version of the image in the white square in the upper panels are shown in the respective lower panels. (D) Representative confocal images and quantification (E) of co‐staining for α‐SMA (*white*) and CD248 (*green*) with membrane receptors (*red*), including AT1, AT2, PDGFRα, and PDGFRβ in the AA of Ang II+Chol *Cd248* ^+/+^ and *Cd248* ^−/−^ mice. The fluorescence intensity profiles from *green*, *red*, and *blue* (DAPI) channels are shown. (F) mRNA levels of AT1 (*Agtr1*), AT2 (*Agtr2*), PDGFRα, and PDGFRβ in Ang II+Chol *Cd248* ^−/−^ and *Cd248* ^+/+^ aorta are shown relative to the mean level in control *Cd248* ^+/+^ aorta, which is set as 1.0 (Con; *N* = 4–5, Ang II+Chol; *N* = 6–9). Scale bars of 50 and 25 µm are indicated, respectively. *p < .05 and **p < .01 by Student's *t*‐test in (B) and (F) and by Mann–Whitney *U* test in (E). Lu: lumen.

Because CD248 deficiency induces a general reduction in several elastin and collagen fibre components, we speculated that CD248 might mediate a signalling pathway critical for fibre component production. Consistent with the literature,^21^ Ang II+Chol upregulated phosphorylation of p38, SMAD2/3, and NF‐κB (Figure S14). The activation of ERK, JNK, AKT, and SMAD2/3 by Ang II+Chol did not differ between *Cd248* ^−/−^ and *Cd248* ^+/+^ aortas. Interestingly, *Cd248* ^−/−^ aorta had significantly lower p38 phosphorylation but higher NF‐κB phosphorylation than *Cd248* ^+/+^ aorta (Figure 5A,B). We further examined the location of p38 downregulation in the aorta of *Cd248* ^−/−^ mice. Immunofluorescence staining showed that phosphorylated p38 was located in the nuclei of cells within the medial layer of *Cd248* ^+/+^ aorta (Figure 5C). However, phosphorylated p38 was barely detected in the nuclei of *Cd248* ^−/−^ aortas. These results suggest that p38 MAP kinase may mediate the loss of CD248 in the regulation of elastin and collagen synthesis in response to Ang II+Chol.

Ang II binds to two different receptor subtypes, AT1 and AT2, and activates many intracellular tyrosine kinases, including p38. These events may be direct or indirect via the activation of tyrosine kinase receptors, including PDGFR. Next, we explored the Ang II or PDGF receptor levels in the mouse aorta. Immunofluorescence staining of the abdominal aorta in *Cd248* ^+/+^ mice showed that ATs, PDGFRs and CD248 were barely detectable in the basal state (Figure S15), whereas Ang II+Chol treatment increased ATs, PDGFRs and CD248 levels in the medial α‐SMA‐positive region (Figure 5D,E). Moreover, co‐localization of CD248 with AT1, PDGFRα or PDGFRβ, but not AT2, was observed in the medial α‐SMA‐positive region of *Cd248* ^+/+^ mice (Figure 5D). CD248 deficiency attenuated the levels of ATs and PDGFRs in the medial α‐SMA‐positive region of the abdominal aorta (Figure 5D,E). The downregulations of AT1 and AT2 proteins are partly contributed by their decreases in mRNA levels in *Cd248* ^−/−^ aorta, whereas the downregulations of PDGFRα and PDGFRβ proteins are not (Figure 5F).

### 3.6 CD248 silencing suppresses the Ang II‐induced response in VSMCs

**FIGURE 6:**
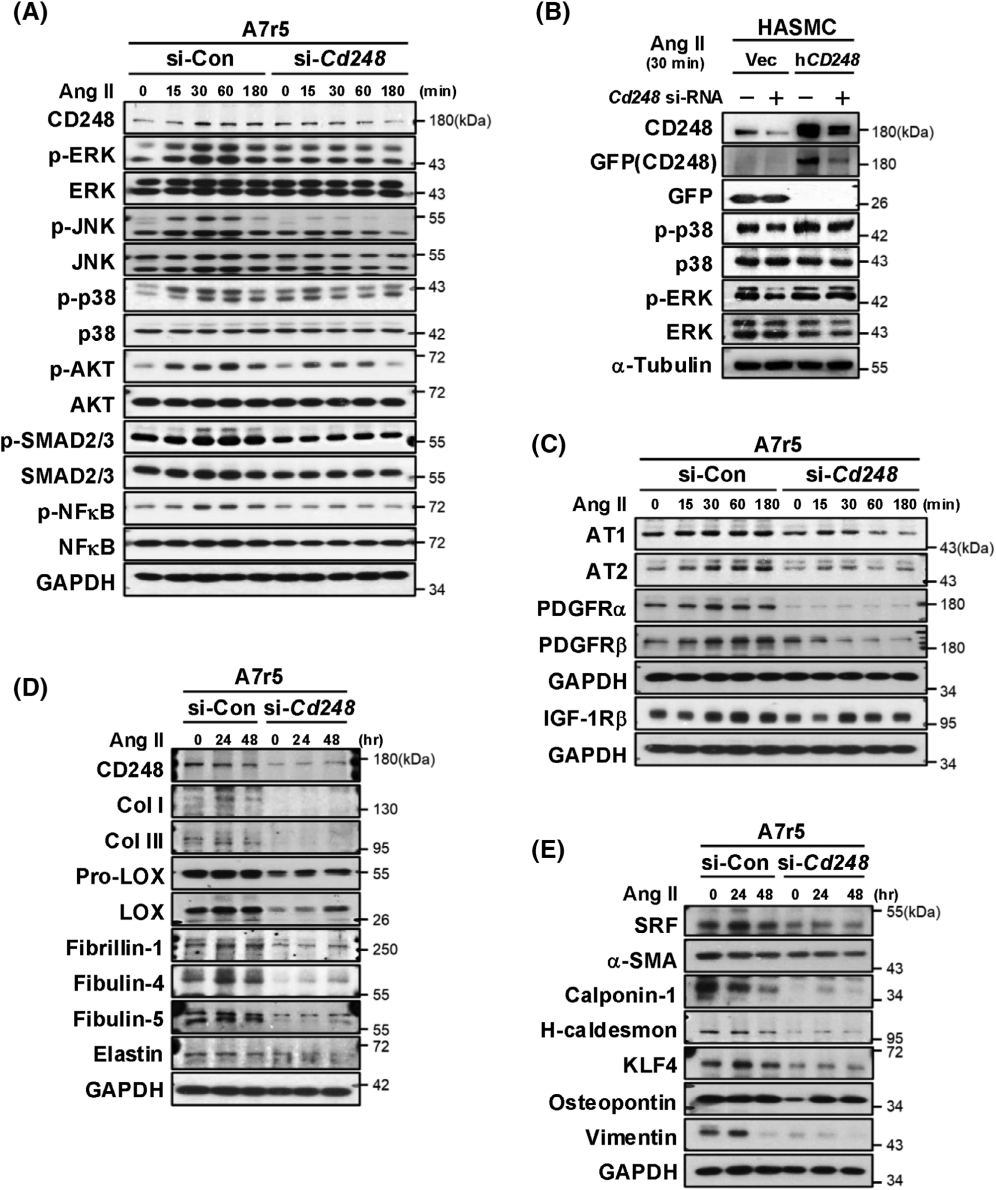
Attenuated Ang II‐induced responses in CD248‐knockdown vascular smooth muscle cells (VSMCs). (A) Time course of phosphorylation of several signalling molecules in response to Ang II (100 nM) treatment in control (si‐Con) and CD248 knockdown (si‐*Cd248*) A7r5 cells. (B) Immunoblot of p38 in response to Ang II (100 nM) treatment at 30 min in CD248‐knockdown human aortic smooth muscle cells (HASMCs) with overexpression of h*CD248* proteins. (C) Immunoblot of Ang II receptors (AT1 and AT2), PDGF receptors (PDGFRα and PDGFRβ), and insulin‐like growth factor 1 receptor beta (IGF‐1Rβ) after Ang II treatment in CD248‐knockdown A7r5 cells. (D) Time course of protein levels of elastin and collagen fibre components in response to Ang II treatment in CD248‐knockdown A7r5 cells. (E) Immunoblot of VSMC‐specific contractile (SRF: serum response factor, α‐SMA, calponin‐1, and H‐caldesmon) and synthetic (KLF4: Krüppel‐like factor 4, osteopontin, and vimentin) biomarkers.

To directly examine the involvement of CD248 in the Ang II signalling pathway, we applied A7r5 and C3H10T1/2 cells to represent VSMCs and fibroblasts within the aorta.^22^ We first examined signalling in A7r5 cells and found that Ang II treatment induced the phosphorylation of ERK, JNK, p38 MAP kinases, and AKT in control cells (Figure 6A and Figure S16A–G). Ang II also modestly induced the phosphorylation of SMAD2/3 and NF‐κB in control cells. However, the upregulated phosphorylation, including ERK, JNK, p38 MAP kinases, AKT, SMAD2/3, and NF‐κB, was significantly attenuated in CD248 knockdown cells. Similarly in human aortic smooth muscle cells, CD248 knockdown decreased phosphorylation of p38 MAP kinase, whereas overexpression of wild‐type human CD248 (h*CD248*) attenuated this downregulation (Figure 6B). These findings further support a direct effect of CD248 in regulation of p38 signalling pathway in VSMCs.

CD248 knockdown also downregulated the protein levels of Ang II receptors (AT1 and AT2) and PDGF receptors (PDGFRα and PDGFRβ) but not IGF‐1Rβ (Figure 6C and Figure S16H–K). These results suggest that CD248 deficiency attenuates Ang II signalling pathway, its receptors AT1 and AT2, and the PDGF receptors PDGFRα and PDGFRβ. We determined the production of elastin and collagen fibre components in A7r5 cells over a longer time course. CD248 knockdown dramatically downregulated collagen fibre components, including Col I, Col III, pro‐LOX and LOX, as well as elastin fibre components, including fibrillin‐1, fibulin‐4, and fibulin‐5 (Figure 6D and Figure S17A–H). These results suggest that CD248 is critical for producing collagen and elastin fibre components in VSMCs.

We tested whether the downregulation of component production by CD248 deficiency was due to a switch in VSMC phenotypes. Our results showed that CD248 deficiency significantly decreased not only contractile phenotypic biomarkers, including SRF, α‐SMA, calponin‐1, and H‐caldesmon but also synthetic phenotypic biomarkers, including KLF4, osteopontin, and vimentin (Figure 6E and Figure S17I–O). Thus, consistent with the in vivo results, CD248 deficiency in the VSMC attenuated both contractile and synthetic phenotypes.

In contrast to VSMCs, CD248 knockdown in C3H10T1/2 cells did not affect Ang II‐mediated signalling, as reflected by normal upregulation of phosphorylated ERK, JNK, p38, AKT, and SMAD2/3 (Figure S18A,C–M) and production of collagen and elastin fibre components (Figure S18B,N–V). Consistently, the Ang II and PDGF receptor levels did not differ between the si‐Con and si‐*Cd248* cells. These data suggest that, specifically in VSMCs but not in fibroblasts, the absence of CD248 attenuates Ang II and PDGF receptors, Ang II‐mediated signalling, and collagen and elastin fibre components.

### 3.7 CD248 silencing inhibits PDGF‐BB‐induced VSMC differentiation, migration, and proliferation

**FIGURE 7:**
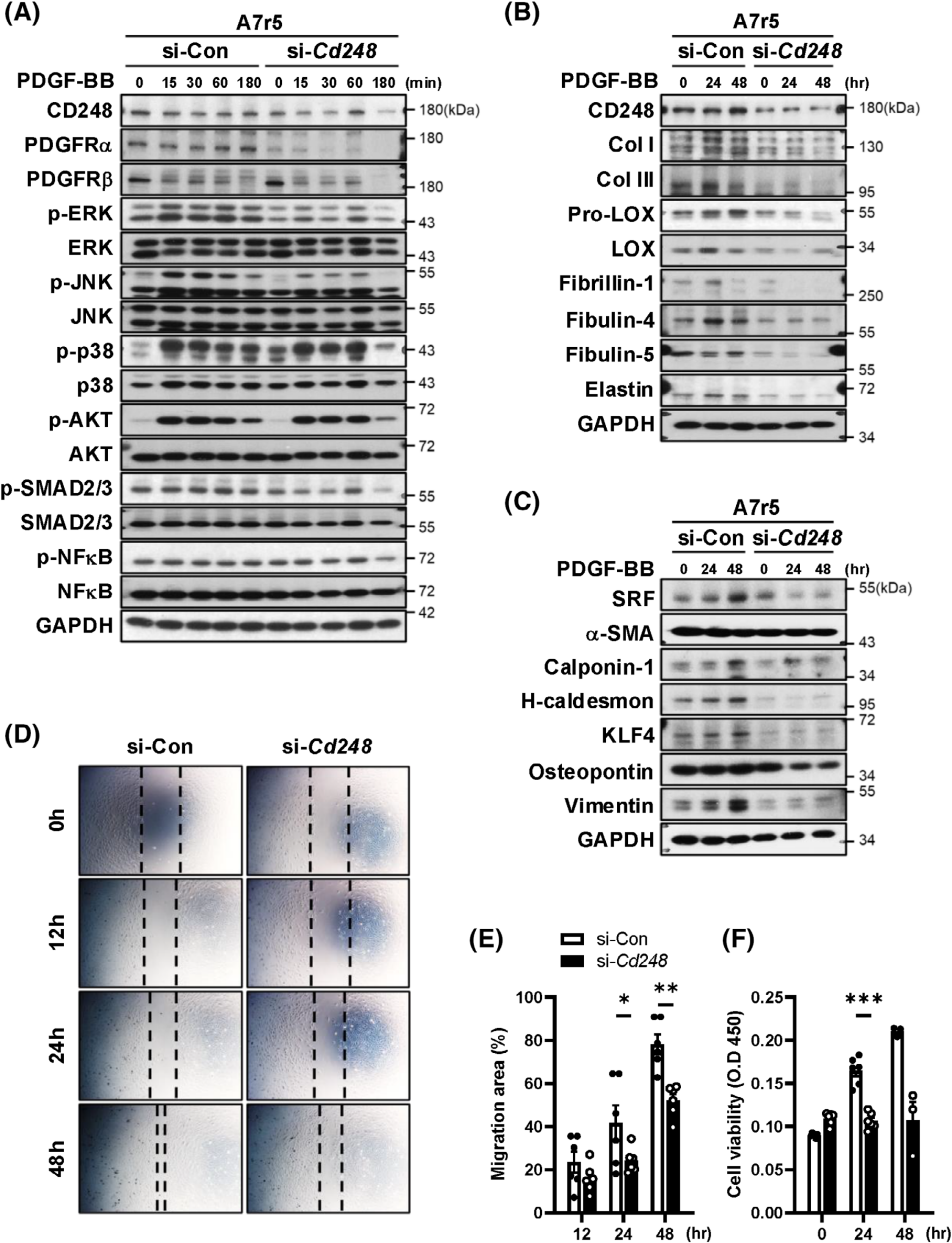
Attenuated PDGF‐induced responses in CD248‐knockdown vascular smooth muscle cells (VSMCs). (A) Time course of phosphorylation of several signalling molecules in response to PDGF‐BB (20 ng/mL) treatment in control (si‐Con) and CD248‐knockdown (si‐*Cd248*) A7r5 cells. (B) Time course of protein levels of elastin and collagen fibre components in response to PDGF‐BB treatment in CD248‐knockdown A7r5 cells. (C) Immunoblot of VSMC‐specific contractile (SRF, α‐SMA, calponin‐1, and H‐caldesmon) and synthetic (KLF4, osteopontin, and vimentin) biomarkers. (D) Representative images of the in vitro scratch‐wound assay after PDGF‐BB treatment for 0, 12, 24, and 48 h. (E) Quantification of the migration area in response to PDGF‐BB for 12, 24, and 48 h from six independent experiments. (F) Cell proliferation in response to PDGF‐BB for 0, 24, and 48 h from three or six independent experiments. *p < .05, **p < .01, and ***p < .001 by two‐way ANOVA with Bonferroni correction.

As our results showed a dramatic downregulation of PDGFRα and PDGFRβ in CD248 knockdown VSMCs, we speculated that the effect of PDGFR activation on cell function might be blunted. We next treated A7r5 cells with PDGF‐BB and examined PDGFRα and PDGFRβ protein levels and downstream signalling. Consistently, CD248 knockdown attenuated PDGFRα and PDGFRβ and blunted downstream responses, including the phosphorylation of ERK, JNK, and p38 (Figure 7A and Figure S19A–I). Furthermore, CD248 knockdown in A7r5 cells significantly attenuated the PDGF‐BB‐mediated induction of collagen fibre components, including Col I, Col III, pro‐LOX, and LOX, and elastin fibre components, including fibrillin‐1, fibulin‐4, and fibulin‐5 (Figure 7B and Figure S20A–H). In contrast, no differences were observed between the si‐Con and si‐*Cd248* groups in C3H10T1/2 cells (Figure S21A–T).

PDGF promotes multiple aspects of synthetic VSMC phenotypes, including the maintenance of differentiation and increases in proliferation and migration.^23^ Consistently, PDGF‐BB treatment increased the expression of both contractile and synthetic phenotypic biomarkers in control cells, whereas the effect of PDGF‐BB was blunted in CD248 knockdown cells (Figure 7C and Figure S20I–O). Moreover, CD248 knockdown attenuated PDGF‐BB‐induced cell migration (Figure 7D,E) and proliferation (Figure 7F). These results reveal a critical role for CD248 in the differentiation, migration, and proliferation of VSMCs in a PDGFR‐dependent manner.

### 3.8 CD248 silencing decreases the protein stability of Ang II and PDGF receptors in VSMCs

**FIGURE 8:**
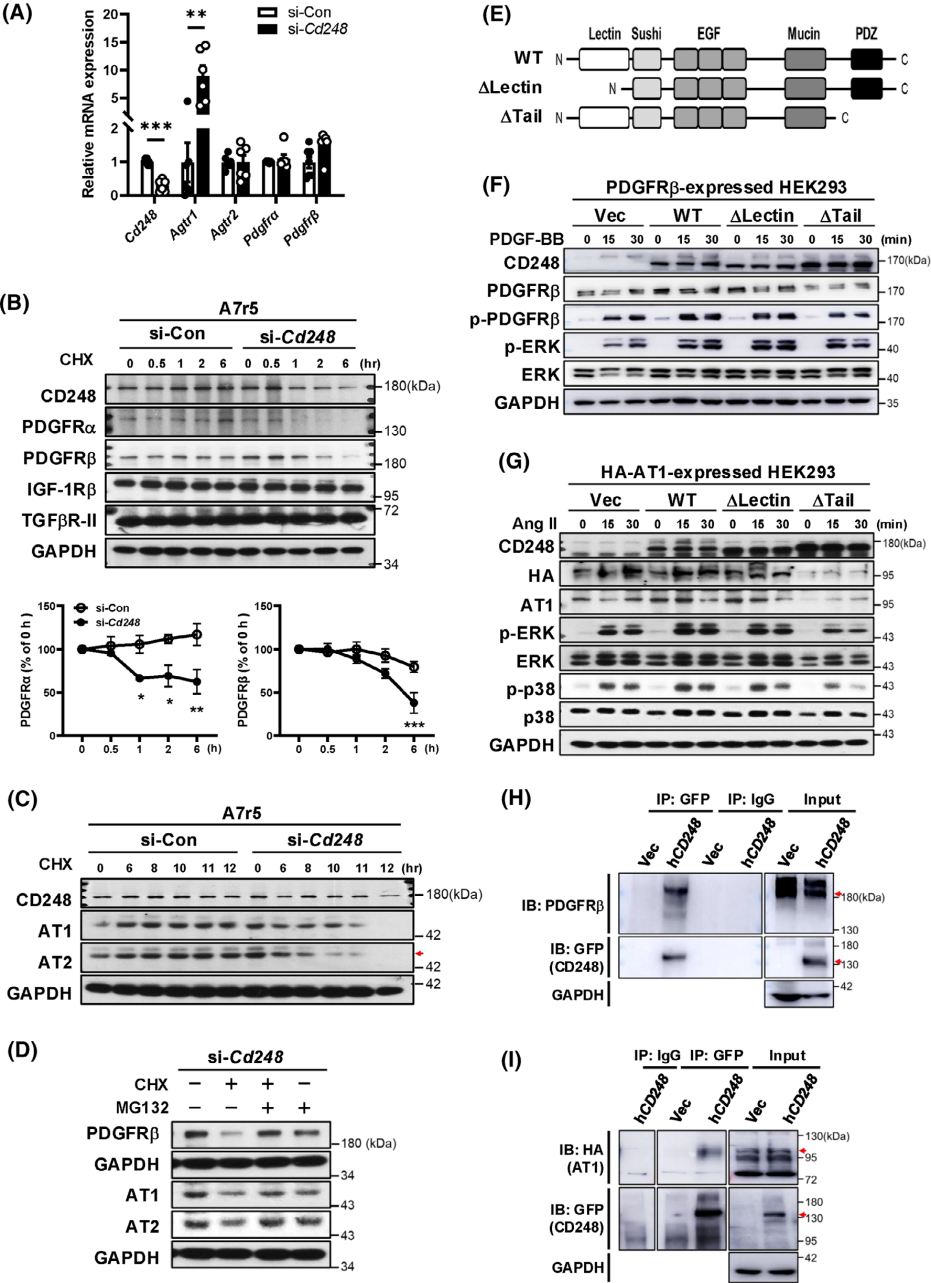
Decreased protein stability of ATs and PDGFRs in CD248‐knockdown vascular smooth muscle cells (VSMCs). (A) mRNA levels of *Cd248*, *Agtr1*, *Agtr2*, *Pdgfrα*, and *Pdgfrβ* in CD248‐knockdown A7r5 cells are shown relative to the mean levels in control cells from six independent experiments. (B) Representative immunoblot and quantification of PDGF receptors (PDGFRα and PDGFRβ), IGF‐1Rβ, and transforming growth factor beta receptor 2 (TGFβR‐II) after cycloheximide (CHX:10 µg/mL) treatment at different time points in control (si‐Con) and CD248‐knockdown (si‐*Cd248*) A7r5 cells. The relative protein ratio to time 0 is calculated from three independent experiments. (C) Immunoblot of Ang II receptors (AT1 and AT2) after CHX treatment in CD248‐knockdown A7r5 cells. (D) Immunoblot of PDGFRβ, AT1, and AT2 in A7r5 cells cultured in the presence of CHX or MG132 for 6 h (PDGFRβ) and 12 h (ATs), respectively. (E) Illustration of CD248 constructs, including intact human CD248 (h*CD248*: WT) and mutants with deletions in the lectin‐like domain (ΔLectin) and cytoplasmic tail (ΔTail). (F) Immunoblot of PDGF downstream signalling molecules in response to PDGF‐BB (20 ng/mL) treatment at different time points in HEK293T cells with overexpression of intact and truncated CD248 proteins. (G) Immunoblot of Ang II downstream signalling molecules in response to Ang II (100 nM) treatment at different time points in HEK293T cells with overexpression of intact and truncated CD248 protein. (H, I) Co‐immunoprecipitation and immunoblot analysis of the interaction between CD248 and PDGFRβ (H) and AT1 (I) in HEK293T cells with overexpression of intact human CD248 (h*CD248*). Anti‐GFP antibodies were used for immunoprecipitation (IP) of GFP‐tagged CD248, and immunoblotting (IB) was performed with PDGFRβ or HA‐tagged AT1. *p < .05, **p < .01, and ***p < .001 by Student's *t*‐test in (A) and by two‐way ANOVA with Bonferroni correction in (B).

Next, we examined the causes of reduced Ang II (AT1 and AT2) and PDGF (PDGFRα and PDGFRβ) receptor expression in CD248‐deficient A7r5 cells. We did not detect decreased expression of these receptors but found increased expression of *Agtr1* in si‐*Cd248* cells (Figure 8A). Using a cycloheximide (CHX) chase assay, we found that CD248 knockdown in A7r5 cells dramatically decreased the protein stability of PDGFRα and PDGFRβ in a shorter time course (Figure 8B), as well as AT1 and AT2 over a longer time course (Figure 8C and Figure S22A–C). However, the protein levels of IGF‐1Rβ and TGFβR‐II did not differ between the groups. MG132 co‐treatment reversed the decreased protein levels of AT1, AT2, and PDGFβ in si‐*Cd248* cells (Figure 8D), suggesting that MG132 attenuates the degradation of these receptors. In contrast, CD248 knockdown in C3H10T1/2 cells did not affect the stability of Ang II or PDGF receptor proteins (Figure S23A,B). These results suggest that CD248 specifically mediates the stability of AT1, AT2, PDGFRα, and PDGFRβ in VSMCs.

### 3.9 The C‐terminal domain is critical for CD248 in mediating the protein stability of Ang II and PDGF receptors

To further dissect which domain in CD248 mediates the protein stability for these membrane receptors, we expressed N‐terminal (C‐type lectin‐like domain; ΔLectin) and C‐terminal cytoplasmic tail (including PDZ domain; ΔTail)‐truncated and full‐length human CD248 (h*CD248*: WT), along with membrane receptors (PDGFRβ or HA‐tagged AT1) in HEK293 cells (Figure 8E). Co‐expression of h*CD248* increased the protein levels of PDGFRβ and intensified PDGF‐BB‐induced signalling, as reflected by the increased phosphorylation of PDGFRβ and ERK (Figure 8F). While deletion of the N‐terminal lectin‐like domain (ΔLectin) did not alter PDGFRβ protein levels and PDGF‐BB‐induced signalling, deletion of the C‐terminal cytoplasmic tail (ΔTail) attenuated them. Similar effects were observed upon co‐expression of HA‐tagged AT1 with truncated and full‐length h*CD248* (Figure 8G). These results suggest that the C‐terminal cytoplasmic tail of CD248 plays a critical role in maintaining membrane receptor protein stability. We then determined if CD248 interacted with PDGF or Ang II receptors by co‐immunoprecipitation assays. Immunoprecipitation of CD248 pulled down PDGFRβ and AT1, respectively (Figure 8H,I). Collectively, these data suggest that CD248 functions as a co‐receptor for PDGFRβ and AT1 through its C‐terminal cytoplasmic tail to maintain the stability of these membrane receptors.

## 4 DISCUSSION

In this study, we reported a dramatic upregulation of CD248 in aortic aneurysms of humans and mice, and showed that genetic deletion of *Cd248* exacerbated Ang II‐induced aortic lesion in mice. Abdominal aorta in *Cd248* ^−/−^ mice exhibited severe disruption of elastic fibres and loss of the VSMC layer. While compensatory ECM deposition was observed in the abdominal aorta of *Cd248* ^−/−^ mice, Col I content and p38 activation were significantly attenuated. Although CD248 was upregulated in both VSMCs and fibroblasts, silencing CD248 resulted in the downregulation of MAP kinase activation and ECM production in VSMCs, but not in fibroblasts. Interestingly, we found that the loss of CD248 in VSMCs destabilized the membrane receptors ATs and PDGFRs and attenuated Ang II‐induced and PDGF‐BB‐induced responses. Finally, we found that the C‐terminal cytoplasmic tail of CD248 plays a key role in mediating the interactions between CD248 and these membrane receptors. Our findings reveal that CD248 regulates the stability of the membrane receptors ATs and PDGFRs in VSMCs to transduce signals for collagen production.

### 4.1 CD248 plays distinct roles in VSMCs and fibroblasts during vascular remodelling

Physiologically, CD248 is highly expressed during embryogenesis, but is greatly reduced in normal tissues after birth. Upregulation of CD248 expression can be found in stromal cells, fibroblasts, pericytes, smooth muscle cells and mesenchymal stem cells,^9^, ^10^ and is associated with tumourigenesis or fibrosis. This study found that CD248 was dramatically upregulated in the medial and adventitial layers of abdominal aortic tissues of patients and mice. We further confirmed that CD248 was induced predominantly in α‐SMA‐positive cells, while its content is lower in the vimentin‐positive cells. Therefore, these results suggest the potential involvement of VSMCs and myofibroblasts in CD248‐mediated AAA pathogenesis.

To test the bona fide nature of CD248 in VSMCs, CD248 was knocked down in A7r5 VSMCs. CD248 knockdown attenuated Ang II‐ and PDGF‐BB‐induced activation of several signalling molecules, inducing ATs, PDGFRs, ERK, JNK, and p38, and the synthesis of collagen and elastic fibre components, including Col I, Col III, LOX, fibrillin‐1, fibulin‐4, and fibulin‐5. Both contractile and synthetic phenotypic markers were markedly downregulated in response to CD248 silencing, along with significant attenuation of PDGF‐BB‐induced proliferation and migration. In contrast, CD248 knockdown in C3H10T1/2 fibroblasts did not affect Ang II‐ and PDGF‐BB‐induced signalling or ECM component production, suggesting that CD248 has a dispensable role in myofibroblast activation. Consistent with others,^9^, ^24^ CD248 could play distinct roles in VSMCs and fibroblasts.

### 4.2 Deletion of CD248 exacerbates Ang II‐induced vascular remodelling with attenuated collagen deposition

Histologically, aortic lesion in *Cd248* ^−/−^ mice showed leukocyte accumulation, elastin disintegration, and VSMC loss. Notably, wild‐type mice exhibited compensatory deposition of Col I between the medial and adventitial layers, and high levels of this protein were detected by immunoblotting in response to Ang II. Surprisingly, CD248 deficiency in mice dramatically attenuated this compensatory effect. Because collagen component proteins provide mechanical stability of the aortic wall,^8^ the loss of compensatory collagen deposition of *Cd248* ^−/−^ abdominal aorta causes structural weakening of the aortic wall, increasing medium‐layer tearing and aneurysmal dilation. Interestingly, we found mRNA levels of the elastin and collagen components showed trends towards downregulation in the basal *Cd248* ^−/−^ aortas (Figure S24A), whereas α‐SMA (*Acta2*) mRNA level was significantly downregulated (Figure S24B–E). Thus, CD248 deficiency in the basal condition may cause modest developmental differences. Upon the pressure overload, the aorta no longer withstands and thus develops aneurysm. Taken together, these results indicate that *Cd248* ^−/−^ aortas with attenuated collagen deposition and reduced vessel wall strength are more vulnerable to pressure overload‐induced dissection.

### 4.3 CD248 deficiency attenuates p38 MAP kinase in VSMCs

To elucidate the mechanism by which CD248 deficiency attenuates ECM production and compensatory collagen deposition during vascular remodelling, we examined several signalling pathways involved in cell growth, migration, and ECM production during vascular remodelling. For example, ERK is involved in Ang II‐augmented growth responses, whereas p38 is critical in Ang II‐induced collagen production in VSMCs.^21^ ERK plays a dominant role in PDGF‐BB‐induced VSMC growth, whereas ERK, JNK, and p38 are involved in VSMC migration.^25^ These results suggest that MAP kinases are involved in vascular remodelling via different mechanisms involving the induction of VSMC growth and collagen production. Interestingly, *Cd248* ^−/−^ abdominal aorta exhibited attenuations in phosphorylation of p38 and translocation to the nucleus within VSMC in the medial layer. In contrast, the phosphorylation of ERK, JNK, AKT, and SMAD2/3 was not altered in *Cd248* ^−/−^ abdominal aorta. Thus, in mice, CD248 deficiency specifically attenuates p38 signalling, which predominantly occurs in VSMCs, and likely mediates compensatory responses in ECM production during vascular remodelling. However, in A7r5 cells, CD248 knockdown significantly attenuated ERK and JNK signalling induced by Ang II and PDGF‐BB. This discrepancy may be attributed to the utilization of different cell sources: adult mouse aortic cells in mice versus embryonic rat VSMCs in A7r5 cells. However, the direct link between p38 MAP kinase signalling and compensatory ECM production in *Cd248* ^−/−^ abdominal aorta during Ang II‐induced vascular remodelling requires further investigation.

### 4.4 The role of CD248 in immune cells

CD248 has been reported to be expressed in immune cells, including macrophages and CD8^+^ T cells. Because we found increased NF‐κB and macrophage infiltration in *Cd248* ^−/−^ AAA, the whole‐body CD248 deficiency is linked to increased inflammation in the AAA. Because of the relatively low expression of CD248 in AAA macrophages, the contribution of CD248 in the macrophage may be modest in the Ang II‐infused AAA model. In addition to macrophages, CD248 is also expressed in CD8^+^ T cells. The increased infiltration of CD248^+^CD8^+^ T cells, characterized by an anti‐inflammatory profile, was found in patients with ascending thoracic aortic aneurysms.^26^ Thus, the crosstalk between inflammatory and vascular cells in vascular remodelling due to CD248 deficiency requires further studies.

### 4.5 Novel role of CD248 in regulating membrane receptor protein stability in VSMCs

Since CD248 is a transmembrane protein, we investigated the mechanism by which CD248 deficiency affects the signalling response induced by Ang II and PDGF‐BB. We first examined the receptor levels of Ang II, AT1, AT2, PDGF‐BB, PDGFRα, and PDGFRβ in CD248 knockdown A7r5 and C3H10T1/2 cells. In A7r5 cells, CD248 deficiency significantly decreased the protein levels of AT1, AT2, PDGFRα, and PDGFRβ, despite normal transcript levels of these membrane receptors (Figure 8A). Consistently, the cycloheximide chase assay confirmed that the protein stabilities of AT1, AT2, PDGFRα, and PDGFRβ significantly decreased in A7r5 cells. However, these effects were not observed in C3H10T1/2 cells. These findings may explain why Ang II‐ or PDGF‐BB‐induced responses were intact in C3H10T1/2 cells, but attenuated in A7r5 cells. CD248 has been shown to be required for efficient PDGFR signalling in the pericyte, VSMC and fibroblast.^9^, ^10^, ^27^ Most evidence has demonstrated the potentiation of signalling reflected by the upregulation of its downstream cascades, ERK and AKT.^13^, ^14^, ^15^, ^28^, ^29^ Although we have previously shown that CD248 plays a critical role in wound healing by enhancing the mitogenic and chemoattractant effects of PDGF‐BB in myofibroblasts, PDGFR protein stability has not been examined.^15^ Since the influence of CD248 on AT receptor protein stability and signalling pathways has not been reported, our findings provide insight into the crosstalk between CD248 and the Ang II signalling pathway.

Next, we investigated the domain of CD248 responsible for the protein stability of these membrane receptors in HEK293T cells. The CD248 protein is composed of a C‐type lectin domain (CTLD), sushi domain, EGF repeats, and a mucin‐like region extracellularly, followed by a transmembrane region and a short cytoplasmic tail containing the PDZ motif.^9^ Interestingly, deletion of the C‐terminal cytoplasmic tail of CD248 downregulated AT1 and PDGFRβ protein levels and the signalling response to Ang II or PDGF‐BB; however, deletion of the N‐terminal CTLD of CD248 had no effect. The cytoplasmic tail of CD248 facilitates PDGF‐BB‐induced cell migration in fibroblasts.^30^ AT1 and AT2 are G protein‐coupled receptors, whereas PDGFRα and PDGFRβ belong to tyrosine kinase receptors. Although both receptor families exhibit distinct domain features, they both can interact with proteins that possess PDZ domains and potentiate downstream signalling.^31^, ^32^ PDZ domains have been shown to associate with both the C‐terminus of membrane receptors to keep them in place.^33^ Without such interactions, the receptors would diffuse out of a specific location on the membrane. Therefore, it is reasonable to speculate that the PDZ domain of CD248 is involved in bridging the membrane receptor to ensure signal transduction and to prevent degradation. The PDZ motif of CD93, another member of the CTLD family, interacts with the Gα adaptor protein, thereby mediating phagocytosis and leukocyte adhesion.^34^ In summary, our results suggest that the C‐terminal cytoplasmic tail acts as a critical domain in mediating the interaction of CD248 with various types of membrane receptors to keep in place and maintain the stability of the protein.

A recent study showed that treatment with recombinant CD248 protein attenuated CaCl_2_‐induced AAA formation in mice.^35^ However, they found that treatment of CD248 recombinant protein promoted phenotypic change and SMAD2 phosphorylation in VSMCs and fibroblasts. Our results also showed that CD248 knockdown in VSMCs attenuated Ang II induced‐SMAD2/3 phosphorylation (Figure 6A and Figure S16F), suggesting that reduced ECM production in *Cd248* ^−/−^ aorta may be related to a general attenuation of Ang II downstream signalling, including SMAD2/3. Moreover, TGFβ protein levels were not altered in CD248 knockout mice and CD248 knockdown cells (Figure S25). The protein stability for TGFβ receptor 2 (TGFBR2) was not changed in CD248 knockdown VSMCs (Figure 8B). Therefore, CD248 seems to affect the protein stability for a specific group of membrane receptors in VSMCs.

Our study has several limitations: Firstly, our study suggests a predominant role of CD248 in VSMCs during Ang II‐induced aortic lesion. CD248 influences the VSMC phenotype during atherosclerotic pathogenesis. Thus, CD248 deficiency favours the contractile phenotype while dampening the synthetic phenotype, contributing to a reduced plaque burden in the *ApoE* ^−/−^ background.^12^ In contrast, our cell and mouse models did not suggest that CD248 deficiency results in a VSMC phenotypic switch. Thus, although CD248 plays distinct roles in different vascular disease models, VSMC has emerged as a collaborative cell type for CD248. Further studies in VSMC‐specific CD248 knockout mice are warranted to confirm the role of VSMC CD248 in AAA pathophysiology. Second, we applied Ang II infusion in vivo to study the role of CD248 in the vascular remodelling. However, some features of Ang II‐induced aneurysms may not be quite typical with those of human AAA.^36^ In the future, it is worth comparing the effects of CD248 deficiency in other animal models for progressive AAA, such as elastase plus BAPN model,^37^ with the Ang II infusion model to study their consequences via different insults.

## 5 CONCLUSIONS

Our findings underscore the critical role of CD248 in VSMCs during the development of Ang II‐induced vascular remodelling. In VSMCs, CD248 stabilizes the protein stability of several membrane receptors, such as ATs and PDGFRs, to potentiate their signal transduction. This interaction is mediated by the cytoplasmic tail of CD248. In Ang II‐induced pressure overload and vascular remodelling, CD248 deficiency attenuates p38 signalling and collagen production, resulting in weakened aortic wall support and an increased risk of aortic dissection. Thus, the increased expression and stability of CD248 in the plasma membrane, as well as the potentiation of its downstream signalling, highlight a potential therapeutic approach for aortic aneurysm.

## AUTHOR CONTRIBUTIONS

Tai‐Tzu Hsieh conceptually designed the strategy for this study and wrote the original draft. Tai‐Tzu Hsieh, Ya‐Chu Ku, and Chu‐Jen Chen performed the experiments and analysed the data. Cheng‐Hsiang Kuo, Bi‐Ing Chang, and Chien‐Hung Yu provided the dissecting microscope and truncated human recombinant CD248 concept and technical assistance. Hua‐Lin Wu and Shu‐Wha Lin provided CD248 knockout mice. Yi‐Heng Li, Pei‐Jane Tsai, and Hua‐Lin Wu participated in discussions and provided critical intellectual input. Chwan‐Yau Luo analysed and interpreted the patient data regarding the AAAs. Yau‐Sheng Tsai interpreted the data, provided intellectual input, supervised the studies, and writing review or editing. All authors were involved with acquisition, analysis and interpretation of data and approved the final version of the article.

## CONFLICT OF INTEREST STATEMENT

The authors declare no conflicts of interest.

## ETHICS STATEMENT

All participants provided written informed consent for this study, which was approved by the Institutional Review Board (B‐ER‐108‐284) of National Cheng Kung University Hospital and conformed to the principles of the Declaration of Helsinki. All animal studies were performed according to protocols approved by the Institutional Animal Care and Use Committee of National Cheng Kung University (107155 and 110203).

## Supporting information



Supporting Information

## Data Availability

All data generated or analysed during this study are included in this published article and its Supporting Information files.
