# Supplementary material for: CD248 deficiency promotes angiotensin II‐induced aortic lesion by attenuating receptor stability in smooth muscle cells
Source: Clin Transl Med. 2025 May 25;15(5):e70352. doi: 10.1002/ctm2.70352 (PMC12104565; doi:10.1002/ctm2.70352)
Supplement: Supplementary file 1 — Supporting Information [file CTM2-15-e70352-s001.pdf]

CD248 Deficiency Promotes Angiotensin II-induced Aortic Lesion by Attenuating Receptor Stability in Smooth Muscle Cells

Tai-Tzu Hsieh, Ya-Chu Ku, Chu-Jen Chen, Cheng-Hsiang Kuo, Bi-Ing Chang, Chien-Hung Yu, Yi-Heng Li, Pei-Jane Tsai, Shu-Wha Lin, Hua-Lin Wu, Chwan-Yau Luo, and Yau-Sheng Tsai

Supplementary Materials and Methods

Human subjects and aortic tissue

Human aortic samples were collected during open aneurysmectomy. Samples at least 6 cm in diameter from eight patients with aortic aneurysm, including 3 abdominal aortic aneurysms (AAA), 3 thoracic aortic aneurysms (TAA), and 2 root aneurysms, were used for immunohistochemical staining. Sections of control human aorta were taken from aortic valve surgery (aortic valve three leaflets). Samples were obtained from three patients with severe aortic stenosis without aortic dilation, and verified by the pathologist with non-tumor structures and within normal limits. All participants provided written informed consent for this study, which was approved by the Institutional Review Board (B-ER-108-284) of National Cheng Kung University Hospital and conformed to the principles of the Declaration of Helsinki. The patient information, including age, sex, surgical site of the aneurysms, and the presence of intraluminal thrombus, was de-identified and is included in Table S1 and S2.

Animal models and treatments

We generated *Cd248*-deficient (*Cd248*$^{-/-}$) mice, in which the *LacZ* gene was targeted to the *Cd248* locus to replace *Cd248* and maintained on a C57BL/6JNarl background (obtained from National Laboratory Animal Center, Taiwan).[1] Six to seven-month-old male *Cd248*$^{+/+}$ and *Cd248*$^{-/-}$ littermates were used in all experiments. The following groups were studied: (1) No Ang II infusion in *Cd248*$^{+/+}$ and *Cd248*$^{-/-}$ mice, (2) Ang II (1000 ng/kg/min, A9525, Sigma-Aldrich) infusion[2,3] and high cholesterol diet (1.5 gm cholesterol, D12079Bi, Research Diets) feeding in *Cd248*$^{+/+}$ and *Cd248*$^{-/-}$ mice. Mice at 5 to 6 months of age were anesthetized and subcutaneously implanted with ALZET osmotic minipumps (1004; DURECT Corporation), delivering Ang II for 28 d.[2,3] Mice

were euthanized with anesthetic overdose at 28 days after pump implantation, and aortic tissues were analyzed. Mice were anesthetized by intraperitoneal injection of Zoletil-Rompun mixture (1 ml Zoletil® (50mg/ml) + 0.1ml Rompun® + 3.9 ml normal saline), with the dosage of 0.1 ml mixture per 20 g mouse body weight. Mice were housed in a specific pathogen-free barrier facility with the humidity and temperature controlled. All animal studies and anaesthetic and analgesic agents were performed according to protocols approved by the Institutional Animal Care and Use Committee of National Cheng Kung University (107155 and 110203).

Serum measurements

Serum was separated by centrifugation and stored at -80°C. Total cholesterol (CH9745, Randox), free fatty acid (FA115, Randox), triglycerides (TRIGS, Randox), and glucose (GL2614, Randox) levels were determined using respective kits.

Measurements of blood pressure

Blood pressure was measured in conscious mice using the tail cuff blood pressure measurement system (BP-2000, Visitech Systems). The mice were trained for 2 days. Blood pressures were measured 20 times per day for 3 consecutive days. The results were calculated as the average from three trials of five to ten measurements each day for 3 consecutive days.

Aortic lesion assessment

After mice was euthanized, the aortic tree from the aortic root to iliac bifurcation were carefully dissected from surrounding tissue. The thoracic aorta/abdominal aorta with or

without aortic lesion were measured and scored according to our severity grading system. Aortic tissue was collected and embedded in paraffin and cut for further histological and immunohistochemical analysis.

Severity grading of aortic lesion

We defined the severity grading of thoracic aortic lesion/abdominal aortic lesion by maximal external aortic diameter, according to the studies[2,4], and by maximal luminal diameter. Severity of aneurysm was graded on a scale from 0 to 3. Grade 0 (no dilation, or maximal outer diameter less than 1.5 times as normal), Grade 1 (1.5 to 2.0 times as normal), Grade 2 (larger than 2 times as normal without rupture), and Grade 3 (ruptured with death).

Morphological Analysis of Aorta

For histological analyses, tissue was fixed in 4% paraformaldehyde in PBS (mouse) or 10% buffered formalin (human) and embedded in paraffin. The mouse AA was cut into three equal parts, and the main aortic lesion region was applied for sectioning. Sections (4 μm) were cut and stained with hematoxylin and eosin (H&E) for histological examination, Verhoeff-Van Gieson (VVG, #25089, AstralDiagnostics, Ethos Biosciences) for elastin fiber network and Masson's trichrome (MT) or Picrosirius red (PSR, SRS250, ScyTek) for collagen. All H&E, VVG, and MT stained slides were viewed using a TissueGnostics GmbH FACS-like Tissue Cytometry (TissueFAXS Plus, TissueGnostics). Images were captured from cross-section of aorta and analyzed for elastin break and collagen intensity by Histoquest 4.0 (TissueGnostics). For elastic fiber break was assigned to each VVG stained slide based on the presence and morphology

of the elastic fiber network. The score was based on the entire elastic lamina of the main aortic lesion region. The following grading criteria were used: 0, normal; 1, short fragmented elastic fibers; 2, intermediate between 1 and 3; 3, intermediate between 3 and 5; 4, absent or nearly absent. MT-positive area, expressed as % positive area per aorta or luminal perimeter, was quantified by colorimetric analysis using TissueFAXS Viewer software (TissueGnostics). PSR-positive area, expressed as % positive area in the media, was quantified using ImageJ software.

Immunohistochemistry and Immunofluorescence

Paraffin-embedded abdominal aorta sections were deparaffinized, blocked, and incubated overnight with primary antibodies (Table S3) or isotype control antibody (Figure S1B) in 3% BSA overnight at 4 °C. For immunohistochemistry, secondary antibody staining was done using a TripleStain IHC kit (ab183292, abcam) and detected with DAB, Emerald, Permanent Red, or AEC (ACG500-IFU, ScyTek) substrate-chromogen solution. For immunofluorescence, sections, followed by primary antibody or isotype control antibody (Figure S26), were then incubated with Polymer HRP-conjugated secondary antibodies using Opal™ 4-Color Automation IHC Kit (NEL820001KT). The images were visualized by confocal laser scanning microscopy (FV3000, Olympus), and colocalization was analyzed using EZ-C1 2.30 software (Nikon). CD248-positive cells are calculated as the percentage of CD248-positive cells out of total nucleated cells per luminal perimeter in 5~10 high-power (200X) fields using ImageJ software.

Immunoblot Analysis

The protein extracts were separated by SDS-PAGE, transferred to PVDF membranes,

and incubated with primary antibodies (Table S3) followed by horseradish peroxidase (HRP)-conjugated secondary antibodies (Vector Laboratories) (Table S3). Immunoreactive protein detection was performed with an enhanced chemiluminescence detection system (GE Healthcare).

RNA analysis

Tissue and cellular RNA were extracted using the Rezol Reagent (Protech Technology Enterprise), and mRNA levels were analyzed with real-time quantitative RT-PCR (StepOne; Applied Biosystems) using *Actb, Gapdh,* or *Ubc* as reference gene in each reaction. Sequences of the primers used for real-time PCR assays are shown in Table S4.

Cell culture

A7r5 cells or C3H10T1/2 cells were cultured at 10-cm dish in high glucose DMEM (DMEM-HG) containing 10% fetal bovine serum (FBS). Cells reaching 80-100 % confluence were transfected with 25 nM siRNA (M-061840-00-0005, Dharmacon) by DharmaFECT 1 Transfection Reagent (T-2001-03, Dharmacon) for 72 hours. Thereafter, cells growing to sub confluence were incubated in DMEM-HG containing 0.5% FBS (A7r5 cells) or no serum (C3H10T1/2 cells) for overnight (A7r5 cells) or 6 h (C3H10T1/2 cells), respectively, and stimulated with Ang II (100 nM) or PDGF-BB (20 ng/mL) for indicated times.[5,6] For the protein stability assay, siRNA transfected cells were treated with 10 μg/ml cycloheximide (66819, Sigma-Aldrich), with or without 10 μM MG132 in DMSO for the indicated times.

Protein Extraction

Cells were washed with ice-cold PBS, lysed in RIPA buffer (50 mM Tris-Base, 150 mM NaCl, 1% NP-40, 0.25% sodium deoxycholate, 1 mM EDTA, pH 7.4) supplemented with protease and phosphatase inhibitors. Lysates were collected by scraping, centrifuged at 13,500 × g for 20 minutes at 4 °C, and the supernatants were collected for protein quantification analysis.

Overexpression of human recombinant CD248

The HASMCs were transiently transfected with pEGFP-N1 vector (Vec) or pEGFP-h*CD248* after CD248 expression was knockdown by siRNA. We transfected 1.5×10^5 of HASMC cells with 0.5 μg of DNA plasmids by electroporation. Two pulses of 1475 V/20 sec was delivered using Neon transfection system (MPK5000, Invitrogen, Thermo Fisher Scientific). After recovering for 24 h, the transfected cells were starved with Smooth Muscle Cell Medium (Catalog #6110, ScienCell Research Laboratories) containing 0.5% FBS overnight and then treated with 100 nM Ang II for 30 minutes. The cell lysates were harvested for immunoblot analysis.

Scratch-wound assay

A7r5 cells cultured in 24-well plates were treated with siRNA for 72 h and treated with PDGF-BB (20 ng/mL) before the scratch assay. A scratch of the cell monolayer was created by a p1000 pipet tip, and photographed at 0, 12, 24, and 48 h after wounding. The closure area of the wound was calculated as follows: migration area (%) = $(A_0 – A_n)/ A_0\times100$, where A_0 represents the area of the initial wound and A_n represents the remaining area of wound at the metering point.[7]

Proliferation assay

The Cell Counting Kit-8 (CCK-8) (96992, Sigma-Aldrich) assay was used to evaluate cell proliferation. A7r5 cells were transfected with siRNA. Selected stable 2000 cells were seeded in a 96-well plate with 100 µl of DMEM-HG containing 0.5% FBS per well, and incubated for overnight, followed by PDGF-BB (20 ng/ml) stimulation for 24 or 48 h. Thereafter, 10 µl of CCK-8 solution was added, and the cells were incubated for 3 h. The absorbance at 450 nm was measured with a microplate reader (340 PC 384, SpectraMax, Molecular Devices). Each experimental group had five duplicate wells, and the experiment was repeated three times.

Generation of PDGFRs and ATs expressed cell lines

To generate the cell lines stably expressing PDGFRβ, we transfected 1 x 10^5 of HEK293 cells with 1 µg of DNA plasmids by electroporation. pCEP4-myc-PDGFRβ was the gift from Erik Procko (Addgene plasmid # 136455).[8] Two pulses of 1150 V/20 sec was delivered using Neon transfection system (MPK5000, Invitrogen, Thermo Fisher Scientific). After electroporation, the cells were allowed to recover for 24 hours, followed by 200 µg/ml Hygromycin selection. The generation of HEK293 cells stably expressing AT1 also followed the same transfected protocol with 400 µg/ml G418 selection. pCXN2-HA-AT1-YFP was the gift from Yusuke Ohba (Addgene plasmid # 101659).[9]

Expression of truncated human recombinant *CD248*

The human *CD248* gene (GenBank NM_020404.3) was cloned into the EcoR I-Kpn I sites of pEGFP-N1 vector, named as pEGFP-h*CD248* (WT). To generate the C-type lectin-like domain deletion (ΔLectin) and intracelluar C-terminus domain (ΔTail) deletion of *CD248*, we cloned the CD248 a.a. 1-29 and 157-757 sequence (ΔLectin,) and CD248 a.a. 1-712 sequence (ΔTail) into pEGFP-N1 vector, respectively.[10] These constructs were named as pEGFP-h*CD248*-ΔLectin and pEGFP-h*CD248*-ΔTail. As previously described, the HEK293 cells stably expressing PDGFRβ were transiently transfected with pEGFP-N1 vector, pEGFP-h*CD248*, pEGFP-h*CD248*-ΔLectin or pEGFP-h*CD248*-ΔTail plasmids. After recovering for 24 h, the transfected cells were starved with DMEM-HG containing 0.5% FBS overnight, and then treated with 20 ng/mL PDGF-BB for 0, 15 and 30 minutes. Similar protocol was also applied to HEK293 cells stably expressing AT1 and followed by 100 nM Ang II treatment.

Co-immunoprecipitation assay

Immunoprecipitation was performed in the total lysate of HEK293 cells using a lysis buffer containing Protease Inhibitor Cocktail (Sigma-Aldrich). Primary (mouse anti-GFP, 66002-1-Ig, Proteintech) and IgG control (1 μg) antibodies were incubated with total protein lysate (250 μg) overnight at 4 °C, and then incubated with protein A/G magnetic beads (MedChemExpress) for 3 h at 4 ◦C. After washing with PBST buffer (0.1% Tween-20) and heating with 95 ◦C for 10 min, the tube was placed on the magnet and the supernatant was analyzed by immunoblotting.

Data analysis

Values are reported as mean ± SEM. Statistical analyses were conducted by Student's

t-test for parametric data and Mann–Whitney *U* test for nonparametric data, and one-way ANOVA or two-way ANOVA with time and genotype as factors followed by Bonferroni correction. The analyses of aortic lesion incidence and severity grading are conducted by Pearson's chi-squared (χ^2) test. Statistical significance was set at P value < 0.05.

References

1. Huang HP, Hong CL, Kao CY*, et al.* Gene targeting and expression analysis of mouse Tem1/endosialin using a lacZ reporter. *Gene Expr Patterns* **11**, 316-326 (2011).
2. Daugherty A, Cassis LA, Lu H. Complex pathologies of angiotensin II-induced abdominal aortic aneurysms. *J Zhejiang Univ Sci B* **12**, 624-628 (2011).
3. Cao RY, Amand T, Ford MD, Piomelli U, Funk CD. The Murine Angiotensin II-Induced Abdominal Aortic Aneurysm Model: Rupture Risk and Inflammatory Progression Patterns. *Front. Pharmacol.* **1**, 9 (2010).
4. Alan Daugherty, Michael W. Manning, Cassis LA. Antagonism of AT2 receptors augments Angiotensin II-induced abdominal aortic aneurysms and atherosclerosis. *Br. J. Pharmacol. Chemother.* **134**, 865-870 (2001).
5. Chen Wen-Dong CY-F, LI Xiao-Dong, GAO Ping-Jin. Angiotensin II induces expression of inflammatory mediators in vascular adventitial fibroblasts. *Acta Physiologica Sinica* **67(6)** 603–610 (2015).
6. An SJ, Liu P, Shao TM*, et al.* Characterization and functions of vascular adventitial fibroblast subpopulations. *Cell. Physiol. Biochem.* **35**, 1137-1150 (2015).
7. Huang G, Cong Z, Wang X*, et al.* Targeting HSP90 attenuates angiotensin II-induced adventitial remodelling via suppression of mitochondrial fission. *Cardiovasc. Res.* **116**, 1071-1084 (2020).
8. Park J, Gill KS, Aghajani AA*, et al.* Engineered receptors for human cytomegalovirus that are orthogonal to normal human biology. *PLoS Pathog.* **16**, e1008647 (2020).
9. Inuzuka T, Fujioka Y, Tsuda M*, et al.* Attenuation of ligand-induced activation of angiotensin II type 1 receptor signaling by the type 2 receptor via protein kinase C. *Sci. Rep.* **6**, 21613 (2016).
10. Valdez Y, Maia M, M. Conway E. CD248: Reviewing its Role in Health and Disease. *Curr. Drug Targets* **13**, 432-439 (2012).

Supplementary Figure 1

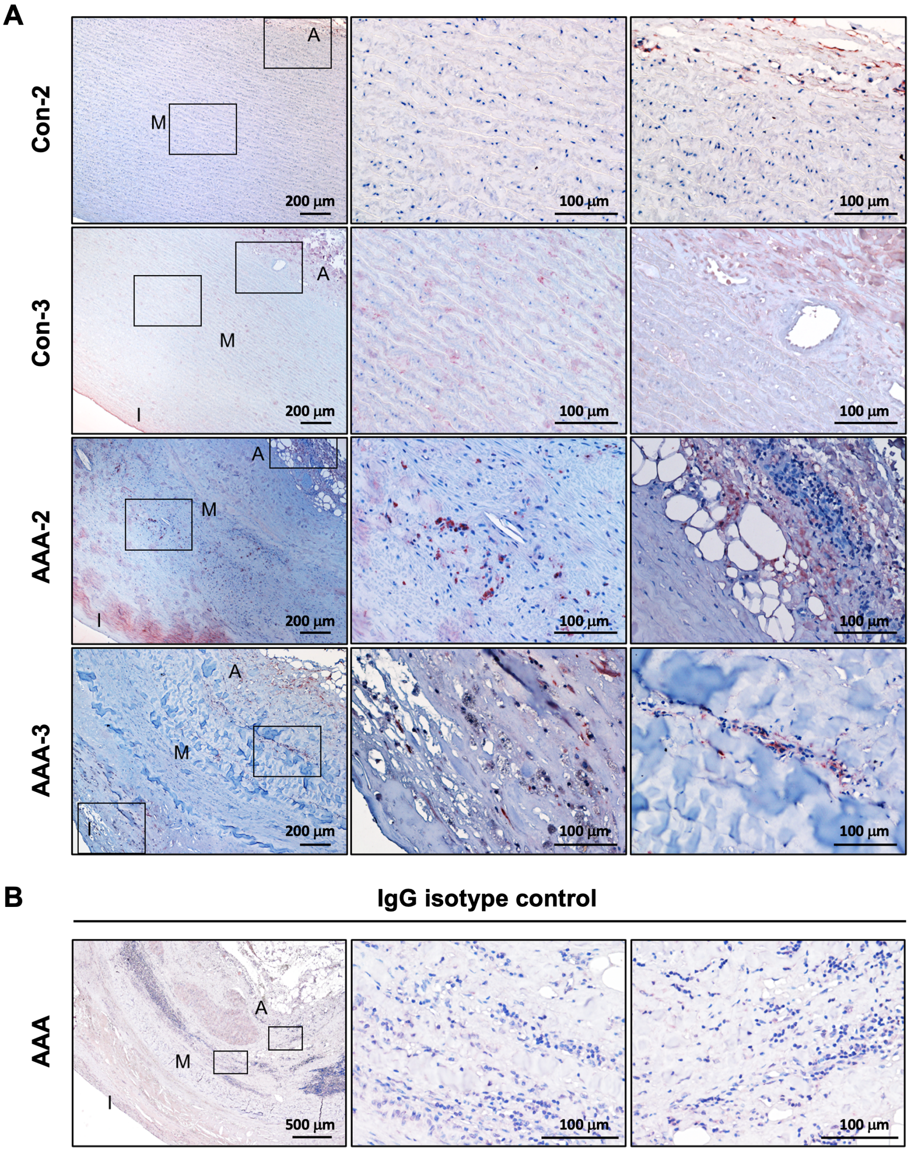

Figure S1

Upregulation of CD248 in human abdominal aortic aneurysm (AAA). A, Representative immunohistochemical staining for CD248 in control human abdominal aortic tissues and AAAs (N=3). I, intima; M, media; A, adventitia. The magnified versions of the images in the black squares in the left panels are shown in the respective right panels. **B,** Representative images for isotype-matched control staining of human AAA. The results showed negative signal for the IgG isotype control. Scale bars are 500 μm, 200 μm, and 100 μm.

Supplementary Figure 2

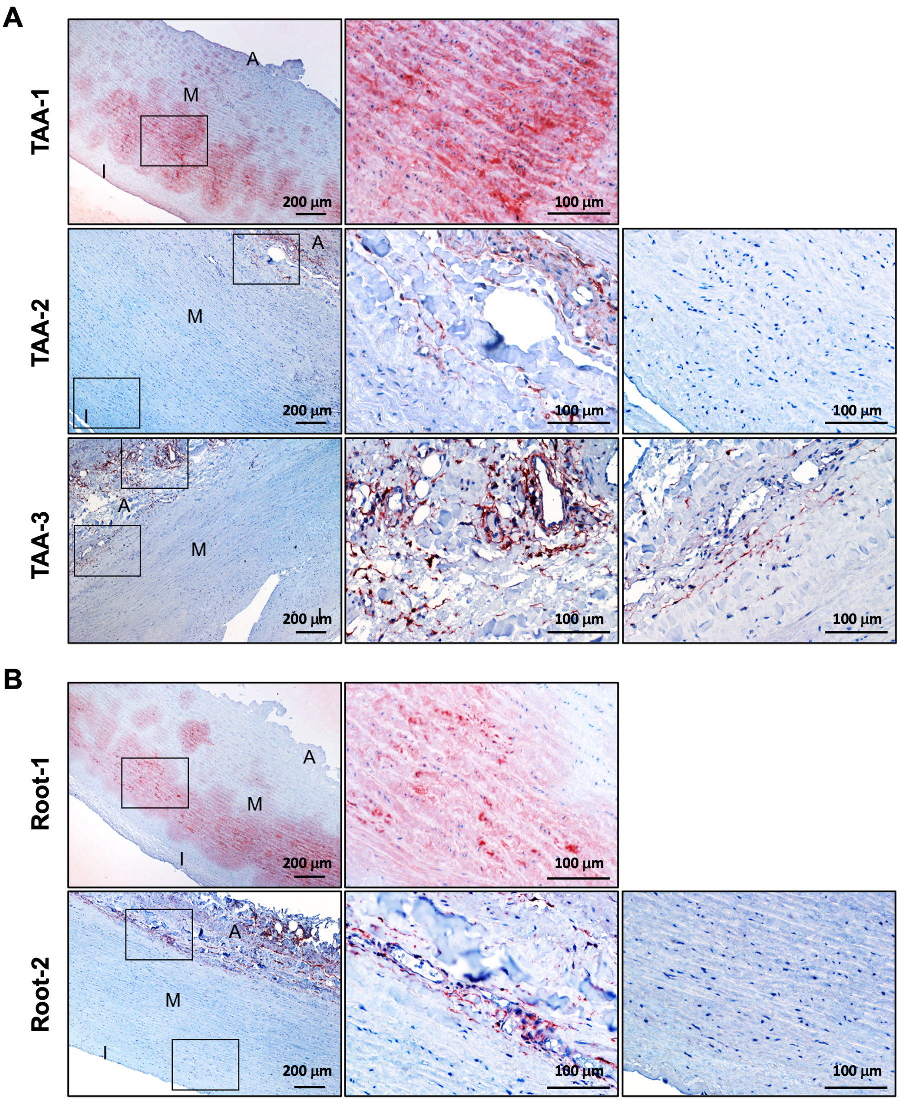

Figure S2

Upregulation of CD248 in human thoracic aortic aneurysm (TAA) and root aneurysm. A, Representative immunohistochemical staining for CD248 in human TAA (N=3). I, intima; M, media; A, adventitia. The magnified versions of the images in the black squares in the left panels are shown in the respective right panels. **B,** Representative immunohistochemical staining for CD248 in human root aneurysm (N=2). Scale bars are 200 µm and 100 µm.

Supplementary Figure 3

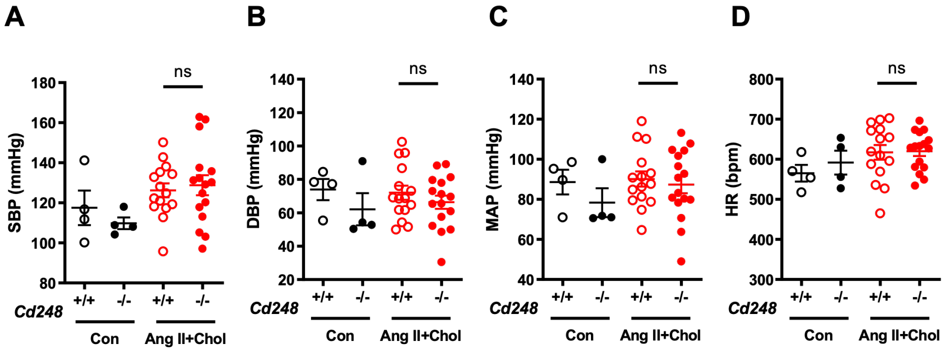

Figure S3

Blood pressure and heart rate measurements in *Cd248*$^{-/-}$ mice. Systolic blood pressure: SBP (**A**), Diastolic blood pressure: DBP (**B**), Mean arterial pressure: MAP (**C**), and Heart rate: HR (**D**) of Ang II+Chol *Cd248*$^{+/+}$ and *Cd248*$^{-/-}$ male mice (Con; N=5, Ang II+Chol; N=15-16). No significance by Student's *t*-test.

Supplementary Figure 4

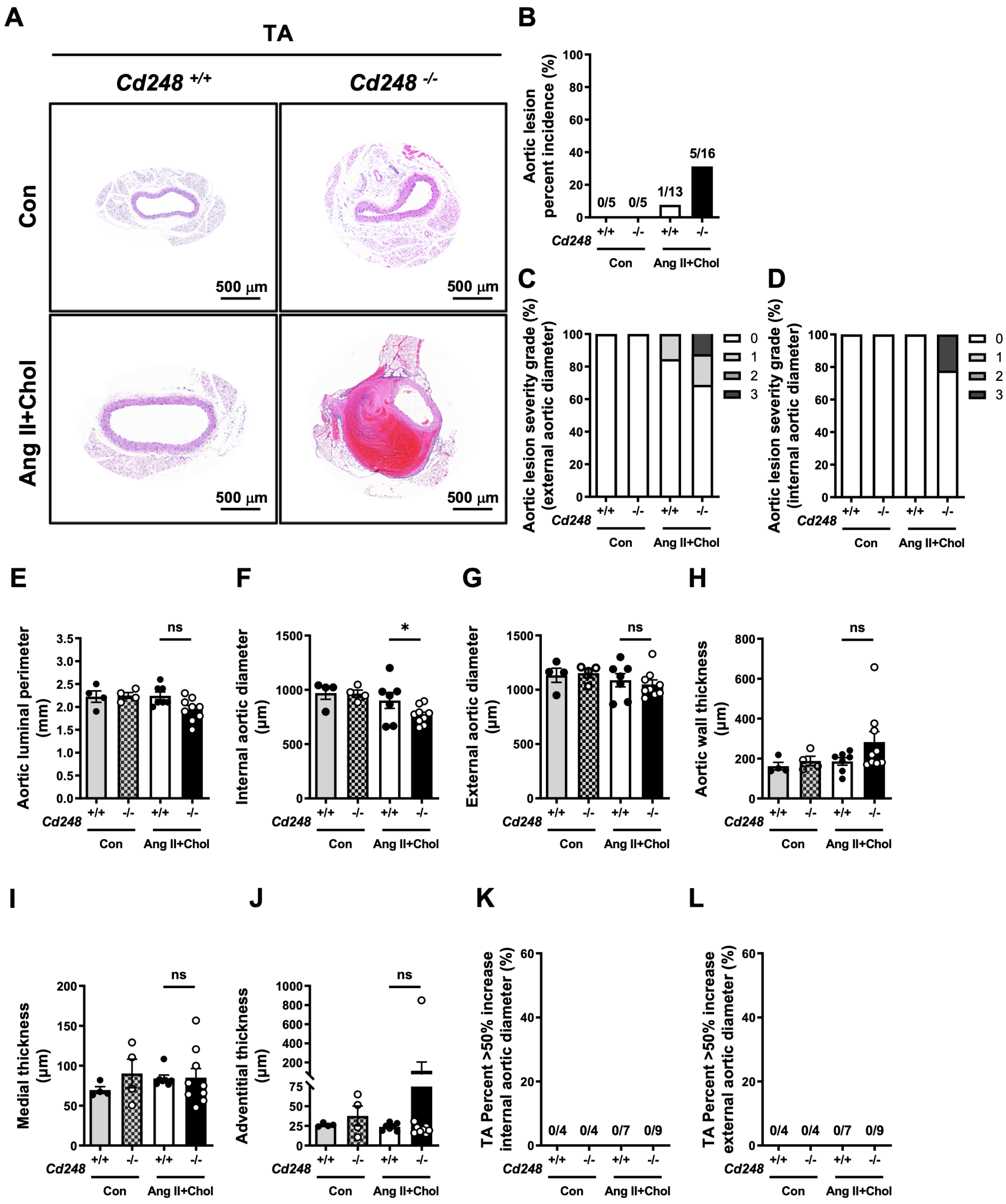

Figure S4

Exacerbated thoracic aortic lesion in *Cd248*$^{-/-}$ mice. A, Representative hematoxylin and eosin (H&E) staining of thoracic aorta (TA). Incidence rate of thoracic aortic lesion (**B**). Percentage of severity grade calculated by maximal external aortic diameter based on the morphology (Con; N=5, Ang II+Chol; N=13-16) (**C**) and by maximal internal aortic diameter based on the histology (Con; N=4, Ang II+Chol; N=7-9) (**D**) in Ang II+Chol *Cd248*$^{+/+}$ and *Cd248*$^{-/-}$ male mice. **E**, Aortic luminal perimeter; **F** and **G**,

Internal aortic diameter and external aortic diameter; **H**, **I**, and **J**, Aortic wall thickness, medial thickness and adventitial thickness; **K** and **L**, The percentage of AA >50% max internal and external aortic diameter in Ang II+Chol *Cd248*$^{+/+}$ and *Cd248*$^{-/-}$ male mice (Con; N=5, Ang II+Chol; N=7-9). Scale bars are 500 μm in **A**. No significance by Pearson's chi-squared (χ^2) test in **B**, **C**, **D**, **K**, and **L**; and *P<0.05 by Student's *t*-test in **E, F, G, H, I,** and **J**.

Supplementary Figure 5

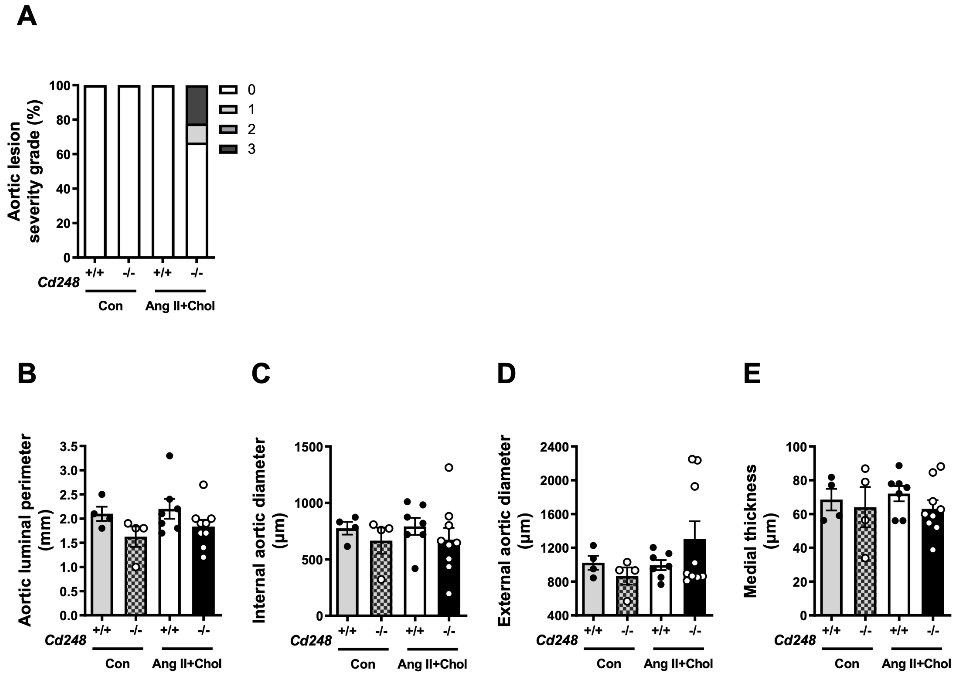

Figure S5

Measurements of abdominal aortic lesion in *Cd248*$^{-/-}$ mice. A, Percentage of severity grade calculated by maximal internal aortic diameter based on the histology; **B,** Aortic luminal perimeter; **C** and **D,** Internal aortic diameter and external aortic diameter; **E,** Medial thickness in Ang II+Chol *Cd248*$^{+/+}$ and *Cd248*$^{-/-}$ male mice (Con; N=4, Ang II+Chol; N=7-9).

Supplementary Figure 6

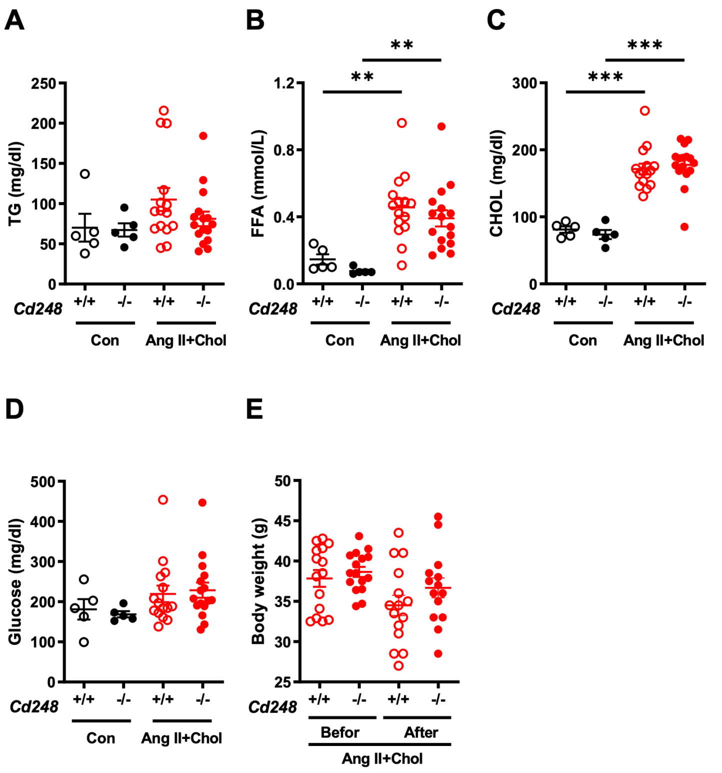

Figure S6

Serum triglyceride, free fatty acid, total cholesterol, and glucose concentrations in *Cd248*$^{-/-}$ mice. The levels of triglyceride: TG (**A**), free fatty acid: FFA (**B**), total cholesterol: CHOL (**C**) and glucose (**D**) in the serum of Ang II+Chol *Cd248*$^{+/+}$ and *Cd248*$^{-/-}$ male mice (Con; N=5, Ang II+Chol; N=15-16). **E,** Body weight of Ang II+Chol *Cd248*$^{+/+}$ and *Cd248*$^{-/-}$ male mice before or after Ang II infusion. ***P*<0.01 and ****P*<0.001 by one-way ANOVA with Bonferroni correction.

Supplementary Figure 7

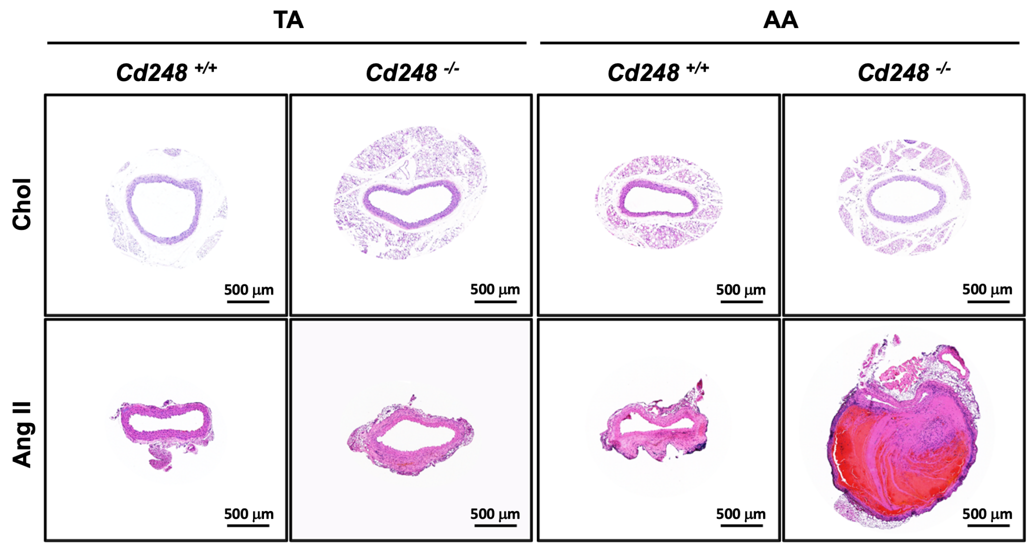

Figure S7

The effects of Ang II treatment and Chol feeding alone on aortic lesion in *Cd248*$^{-/-}$ mice. Upper panels show representative H&E staining of TA (left panels) and AA (right panels) from cholesterol (Chol) fed *Cd248*$^{+/+}$ and *Cd248*$^{-/-}$ male mice (N=4-5). Lower panels show representative H&E staining of TA (left panels) and AA (right panels) from Ang II alone-treated *Cd248*$^{+/+}$ and *Cd248*$^{-/-}$ male mice (N=6-9). Scale bars are 500 µm.

Supplementary Figure 8

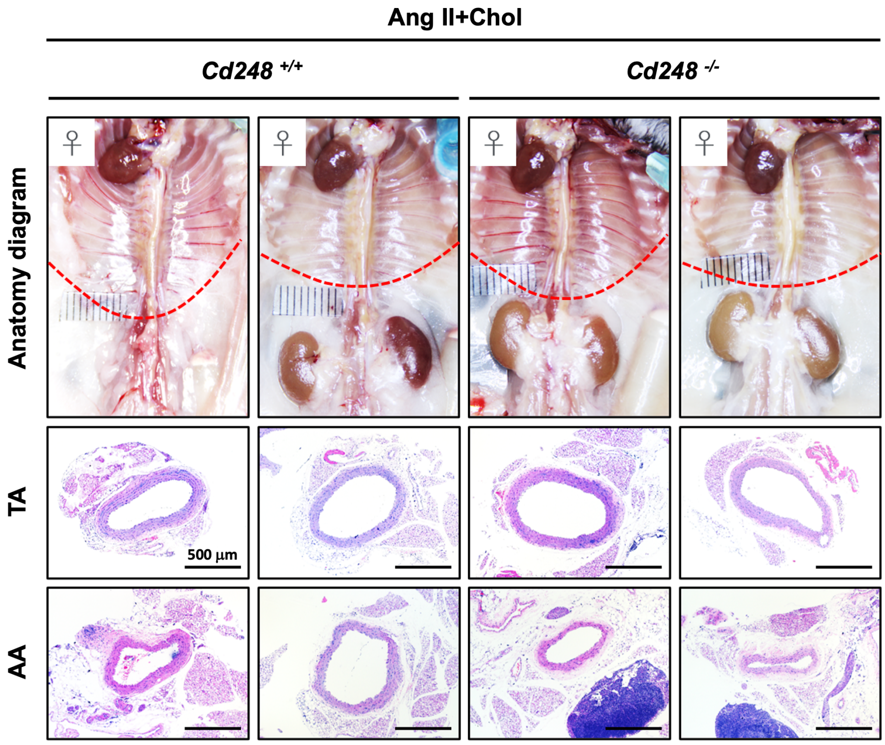

Figure S8

Normal thoracic aorta (TA) and abdominal aorta (AA) in female *Cd248*$^{-/-}$ mice.

Upper panels show representative photographs of the aorta. The red dotted line indicates the diaphragm. Middle panels show H&E staining of TA from Ang II+cholesterol (Chol) fed *Cd248*$^{+/+}$ and *Cd248*$^{-/-}$ female mice (N=2). Lower panels show representative H&E staining of AA. Scale bars are 500 µm.

Supplementary Figure 9

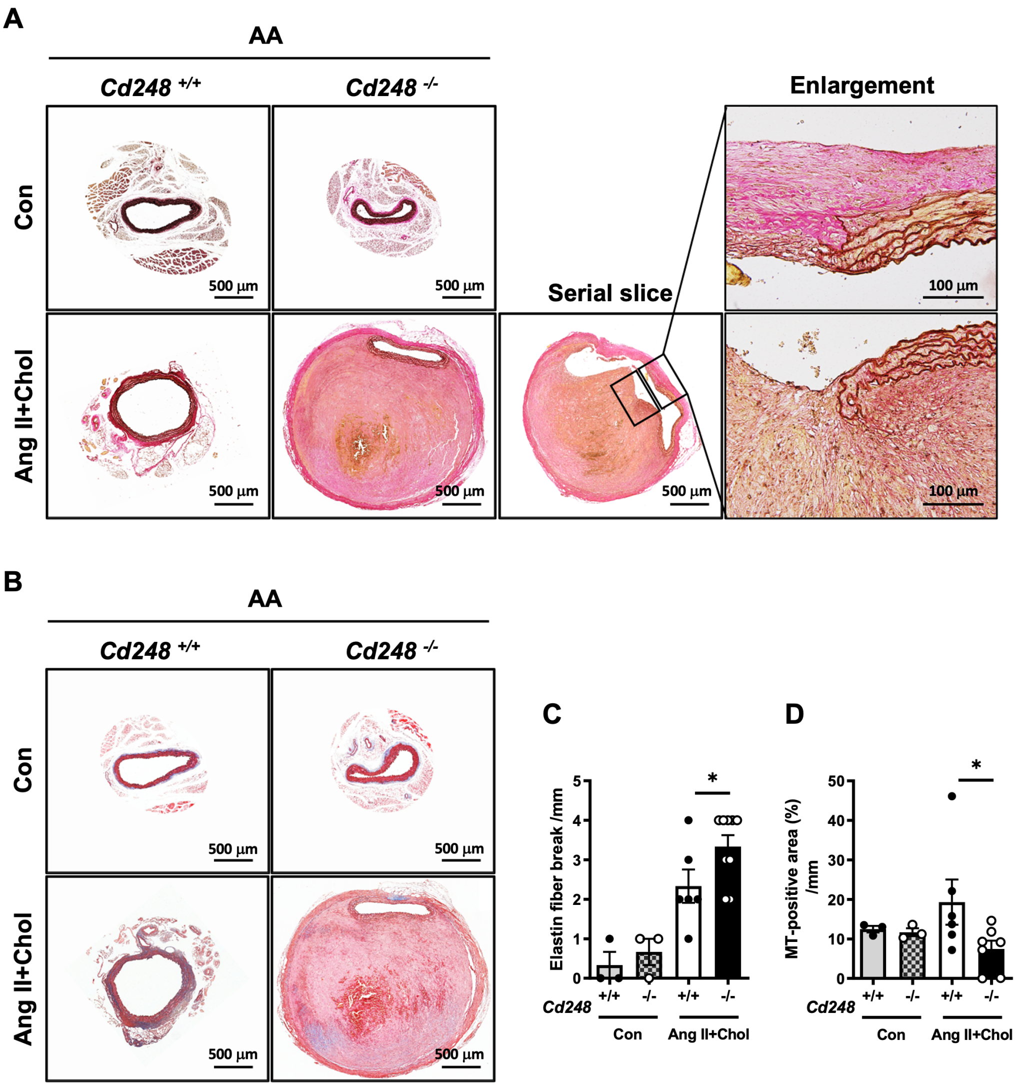

Figure S9

Increased elastic fiber breakage and attenuated collagen deposition in *Cd248*$^{-/-}$ aorta in response to Ang II+Chol. A, Representative Verhoeff-Van Gieson (VVG) and Masson's trichome (MT) (**B**) staining of abdominal aorta to illustrate the extent of elastin fiber break and collagen deposition respectively in *Ang* II+Chol *Cd248*$^{+/+}$ and *Cd248*$^{-/-}$ male mice (Con; N=3, Ang II+Chol; N=6-9). **C,** Quantitation of elastin fiber breakage and collagen-positive area (**D**) normalized to the luminal perimeter (mm). Scale bars of 500 µm and 100 µm are indicated, respectively. **P*<0.05 by Student's *t*-test.

Supplementary Figure 10

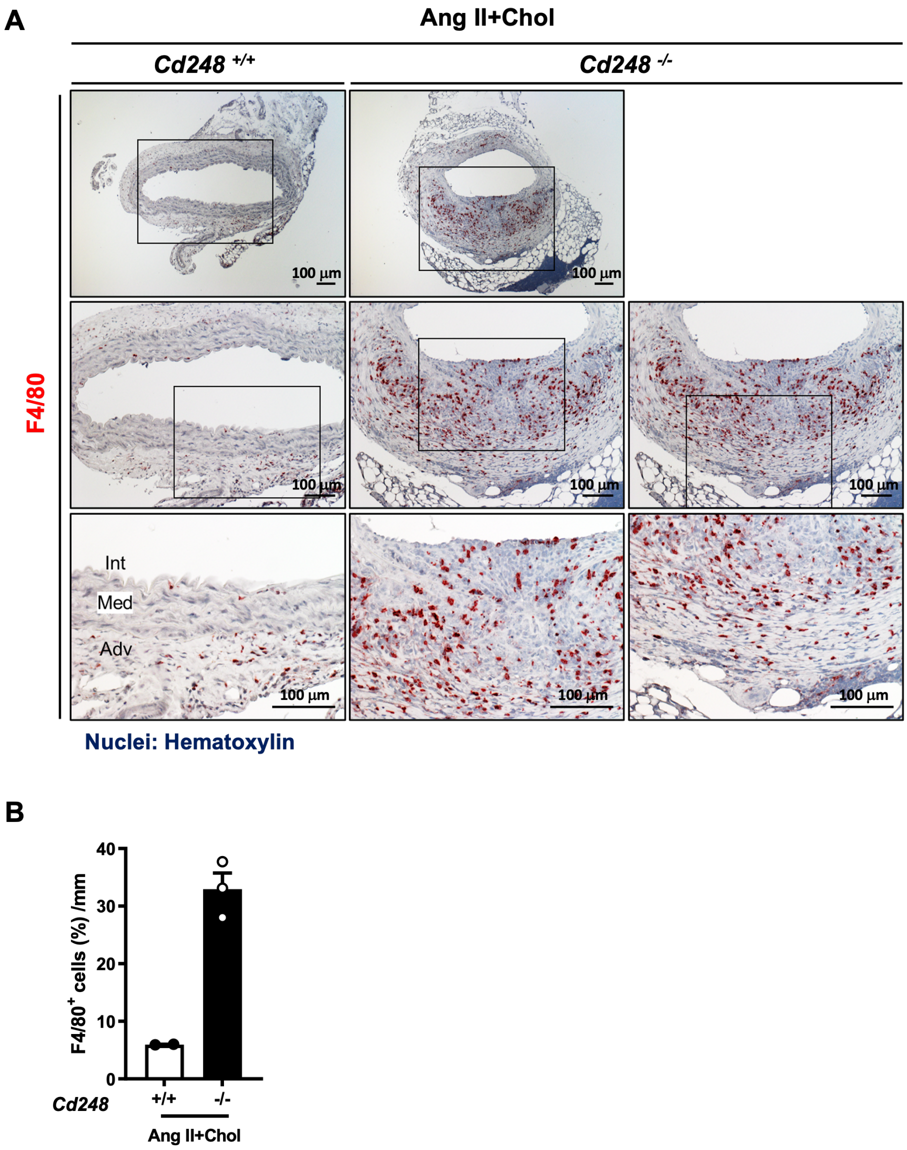

Figure S10

Macrophage infiltration of abdominal aorta (AA) in Ang II+Chol *Cd248*$^{-/-}$ mice. A, Immunohistochemical staining and quantification (**B**) for F4/80 (*red*) of AA in Ang II+Chol *Cd248*$^{+/+}$ (left panel) and *Cd248*$^{-/-}$ (right panels) male mice (N=2-3). *Cd248*$^{+/+}$ aorta exhibits few F4/80 (*red*)-positive cells in the boundary of media and adventitia. *Cd248*$^{-/-}$ aorta exhibits increased F4/80 (*red*)-positive cells. The hematoxylin stains cell nuclei in *purplish blue*. Scale bars are 100 µm in **A**.

Supplementary Figure 11

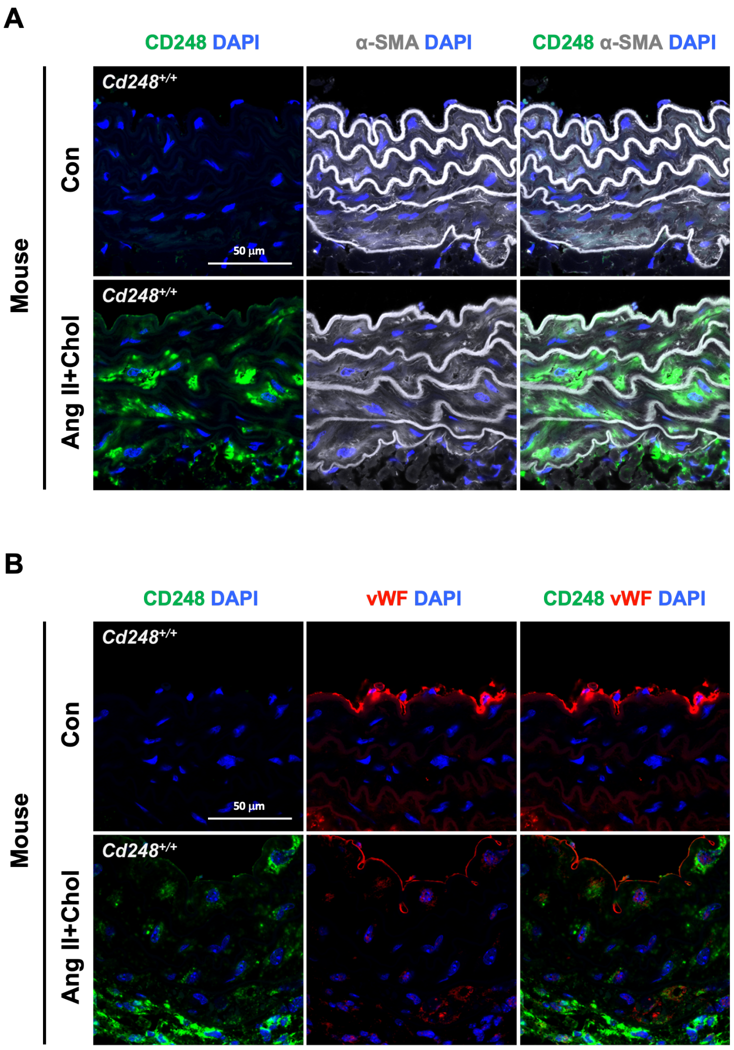

Figure S11

Expression of CD248 in the vascular smooth muscle cell (VSMC) and endothelial cell (EC) of mouse abdominal aorta (AA). A, Representative confocal images of co-staining for CD248 (*green*) with α-SMA (*white*) in the AA of control and Ang II+Chol *Cd248*$^{+/+}$ mice. **B,** Representative confocal images of co-staining for CD248 (*green*) with von Willebrand Factor (vWF, *red*). Scale bars are 50 μm.

Supplementary Figure 12

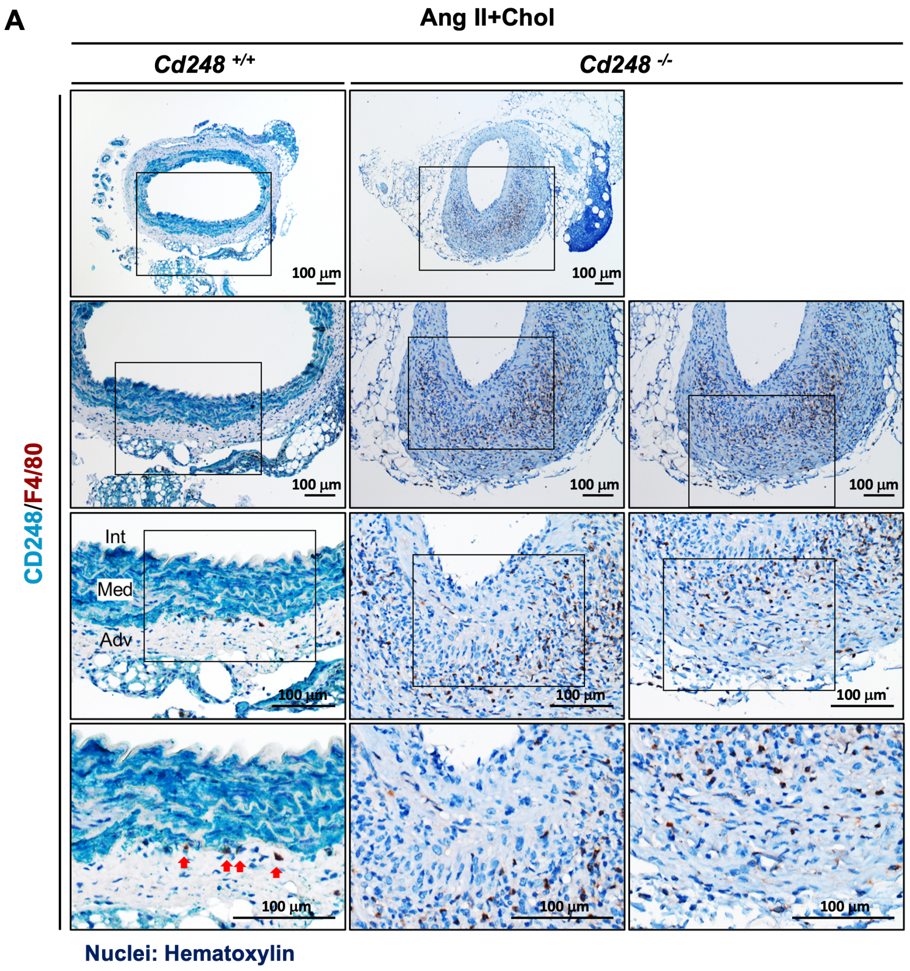

Figure S12

Expression of CD248 in the macrophage of mouse abdominal aorta (AA). A, Immunohistochemical staining for CD248 (*blue*) and F4/80 (*brown*, filled arrows) of AA in Ang II+Chol *Cd248*$^{+/+}$ (left panels) and *Cd248*$^{-/-}$ (right panels) male mice. The magnified versions of the images in the black squares in the upper panels are shown in the respective lower panels. *Cd248*$^{+/+}$ aorta exhibits CD248 (*blue*) expression with very few F4/80 (*brown*)-positive cells in the boundary of media and adventitia. *Cd248*$^{-/-}$ aorta exhibits increased F4/80 (*brown*)-positive cells. The hematoxylin stains cell nuclei in *purplish blue*. Scale bars are 100 µm.

Supplementary Figure 12

B

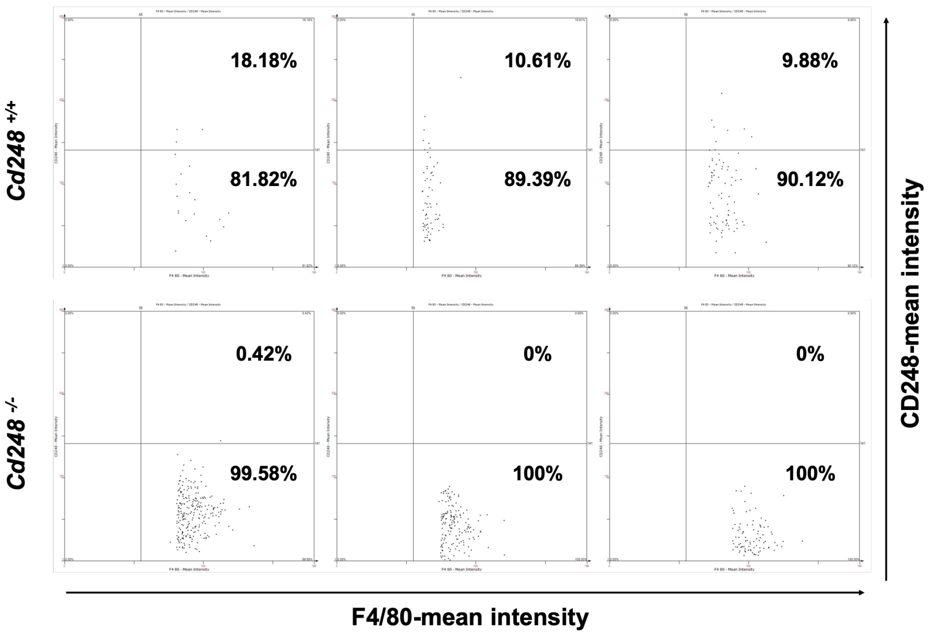

C

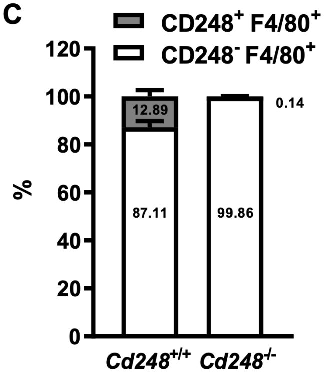

Figure S12 (Continued)

B, Quantification of CD248 (*blue*) expression with F4/80 (*brown*)-positive cells by Histoquest 4.0 (TissueGnostics) and summary bar graph **(C)**.

Supplementary Figure 13

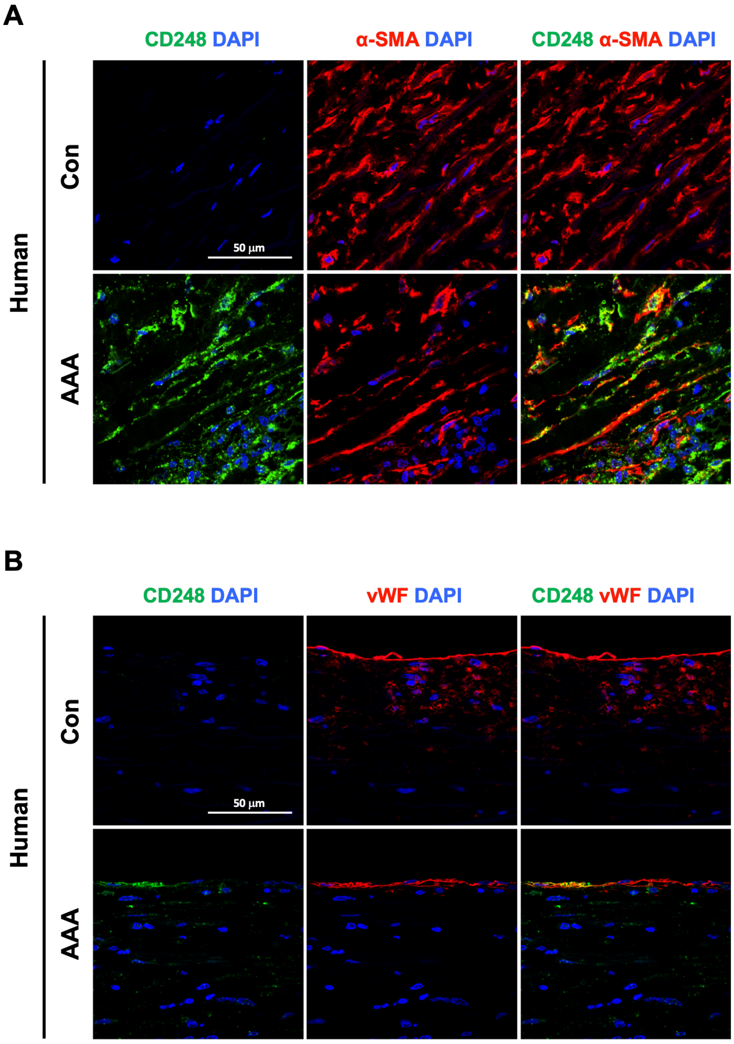

Figure S13

Expression of CD248 in the vascular smooth muscle cell (VSMC) and endothelial cell (EC) in patients with abdominal aortic aneurysm (AAA). A, Representative confocal images of co-staining for CD248 (*green*) with α-SMA (*red*) in control human abdominal aortic tissues and AAAs. **B,** Representative confocal images of co-staining for CD248 (*green*) with von Willebrand Factor (vWF, *red*). Scale bars are 50 μm.

Supplementary Figure 14

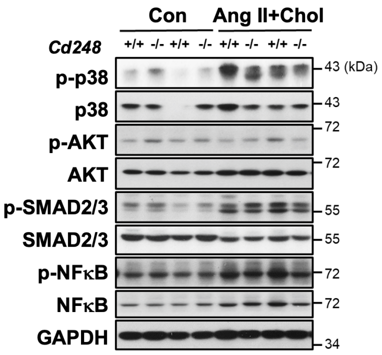

Figure S14

The effect of Ang II on its downstream signaling molecules in *Cd248*$^{+/+}$ and *Cd248*$^{-/-}$ aorta. Representative immunoblot of Ang II downstream signaling molecules in the AA of control and Ang II+Chol mice.

Supplementary Figure 15

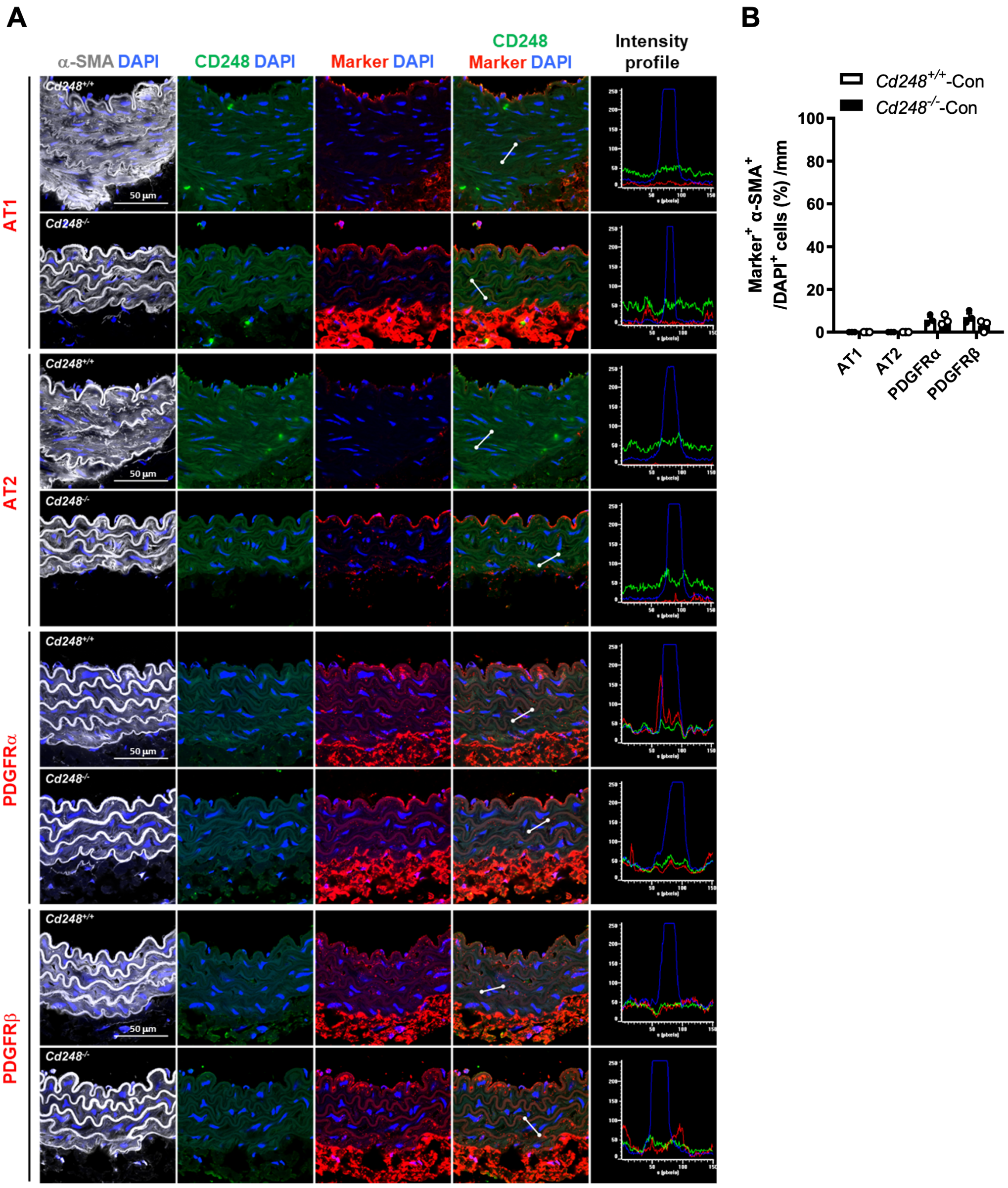

Figure S15

Low expression of Ang II and PDGF receptors in the abdominal aorta (AA) from basal *Cd248*$^{+/+}$ and *Cd248*$^{-/-}$ mice. A, Representative confocal images and quantification (**B**) of co-staining for α-SMA (*white*) and CD248 (*green*) with membrane receptors (*red*), including AT1, AT2, PDGFRα, and PDGFRβ in the AA of Ang II+Chol *Cd248*$^{+/+}$ and *Cd248*$^{-/-}$ male mice. The fluorescence intensity profiles from *green*, *red*, and *blue* (DAPI) channels are shown. Scale bars are 50 μm in **A**. No significance by Mann–Whitney *U* test.

Supplementary Figure 16

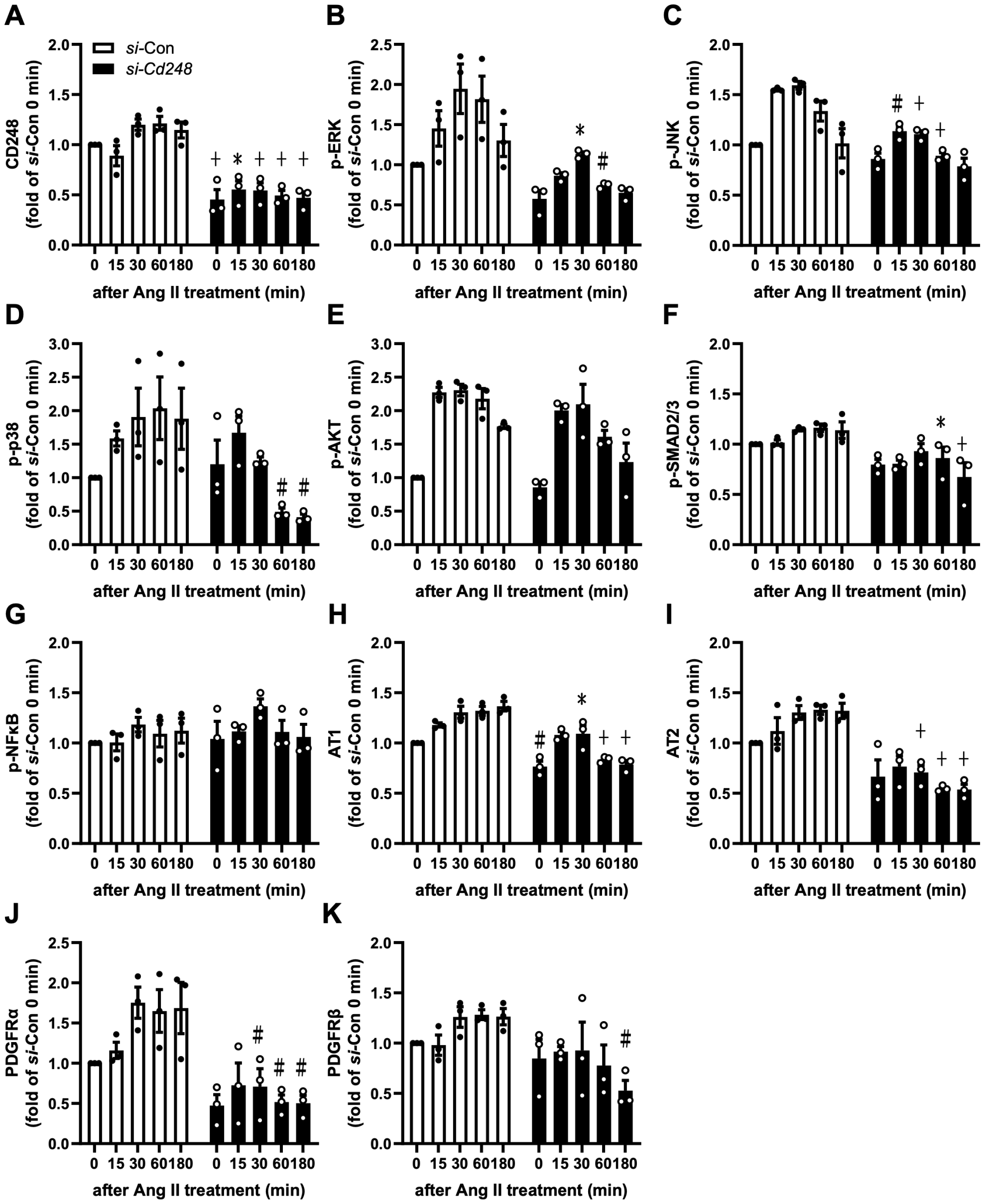

Figure S16

Quantification for western blot images in Figure 6A and 6C. A-G, Quantification for the immunoblots of the time course of phosphorylation of several signaling molecules in response to Ang II (100 nM) treatment in control (si-Con) and CD248 knockdown (si-*Cd248*) A7r5 cells as shown in Figure 6A (N=3). **H-K**, Quantification for the immunoblots of Ang II receptors (AT1 and AT2) and PDGF receptors (PDGFRα and PDGFRβ) after Ang II treatment in CD248-knockdown A7r5 cells as shown in Figure 6C (N=3). *P<0.05, $^{\#}P$<0.01, and ^{+}P<0.001 by two-way ANOVA with

Bonferroni correction.

Supplementary Figure 17

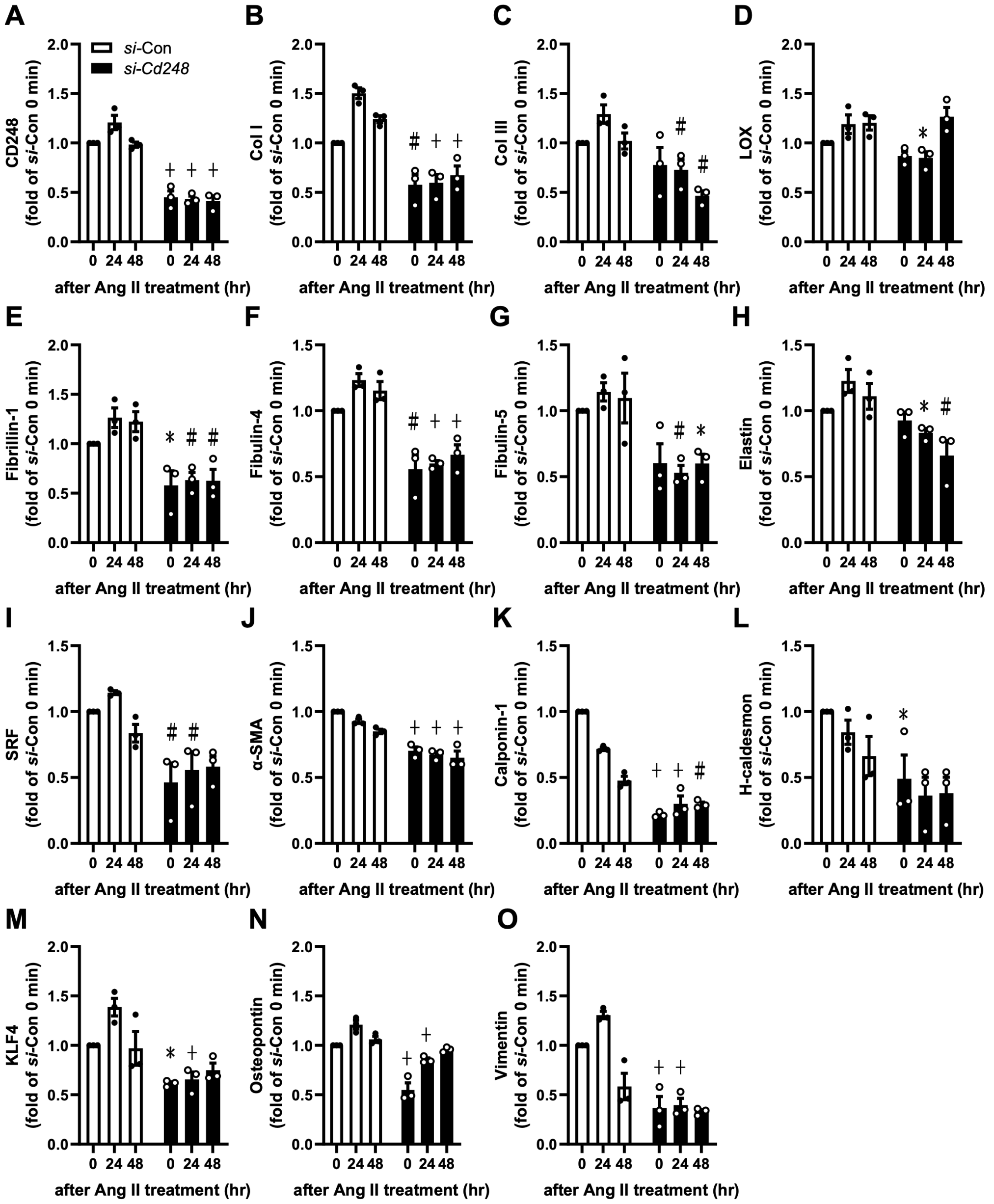

Figure S17

Quantification for western blot images in Figure 6D and 6E. A-H, Quantification for the immunoblots of the time course of protein levels of elastin and collagen fiber components in response to Ang II treatment in CD248-knockdown A7r5 cells as shown in Figure 6D (N=3). **I-O,** Quantification for the immunoblots of VSMC-specific contractile and synthetic biomarkers as shown in Figure 6E (N=3). $^{*}P<0.05$, $^{\#}P<0.01$, and $^{+}P<0.001$ by two-way ANOVA with Bonferroni correction.

Supplementary Figure 18

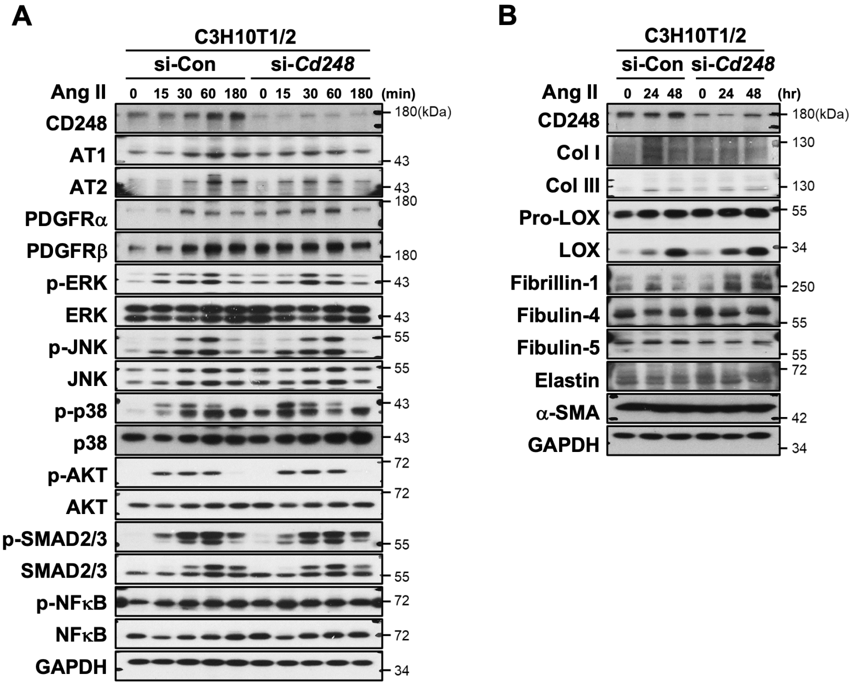

Figure S18

Normal Ang II-induced responses in CD248-knockdown fibroblasts. A, Time course of phosphorylation of several signaling molecules in response to Ang II (100 nM) treatment in control (si-Con) and CD248-knockdown (si-*Cd248*) C3H10T1/2 cells. **B,** Time course of protein levels of elastin and collagen fiber components and α-SMA in response to Ang II treatment in CD248-knockdown C3H10T1/2 cells.

Supplementary Figure 18

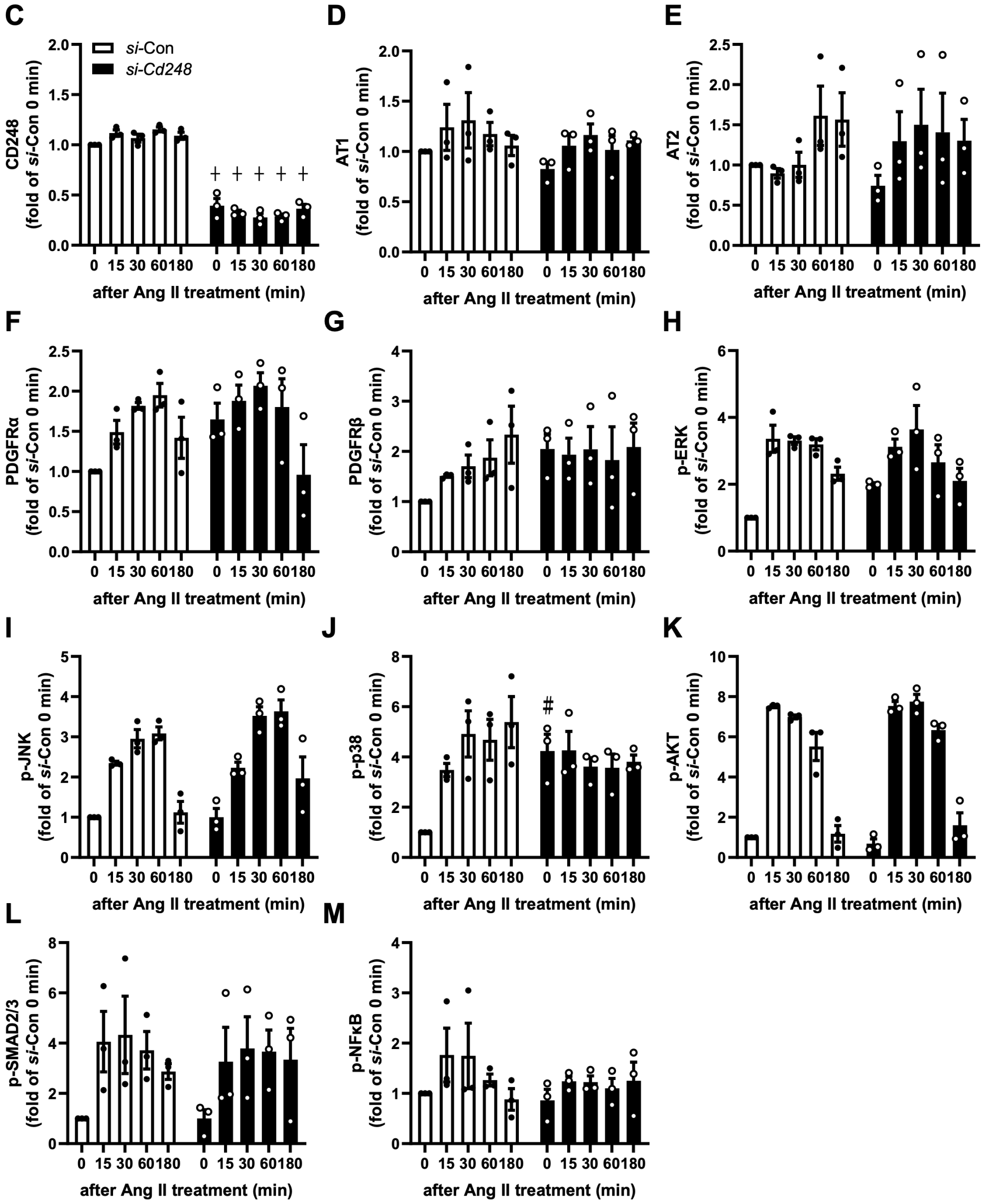

Figure S18 (Continued)

C-M, Quantification for the immunoblots of the time course of phosphorylation of several signaling molecules in response to Ang II (100 nM) treatment in control (si-Con) and CD248-knockdown (si-*Cd248*) C3H10T1/2 cells as shown in Figure S18A (N=3). $^{\#}P$ <0.01 and $^{+}$ P<0.001 by two-way ANOVA with Bonferroni correction.

Supplementary Figure 18

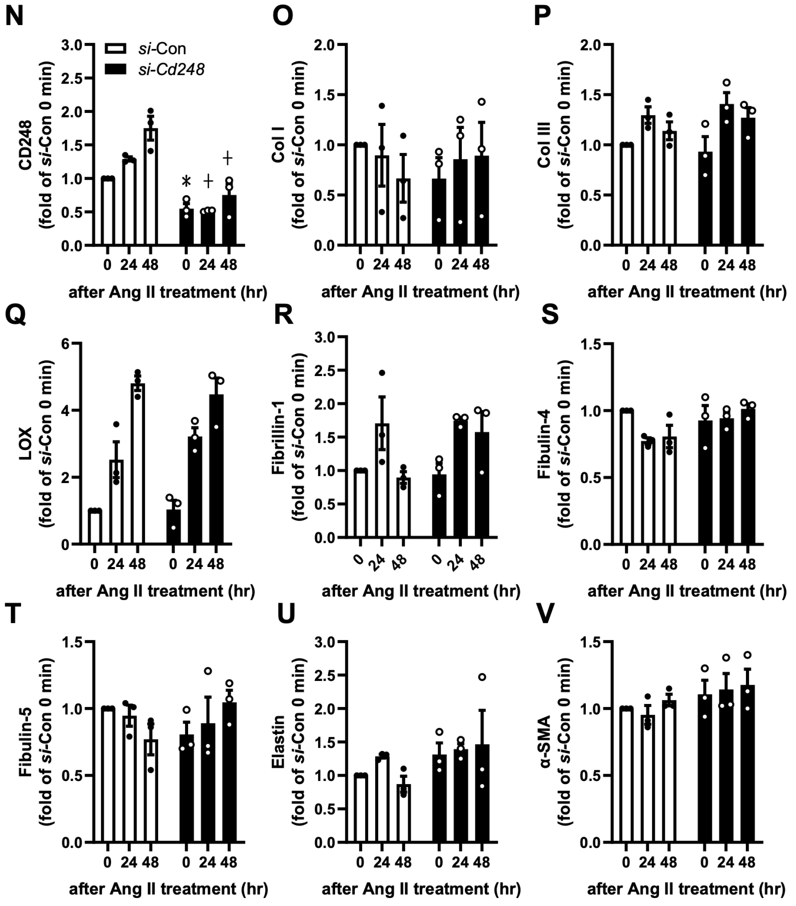

Figure S18 (Continued)

N-V, Quantification for the immunoblots of time course of protein levels of elastin and collagen fiber components and α-SMA in response to Ang II treatment in CD248-knockdown C3H10T1/2 cells as shown in Figure S18B (N=3). *P<0.05 and ^{+}P<0.001 by two-way ANOVA with Bonferroni correction.

Supplementary Figure 19

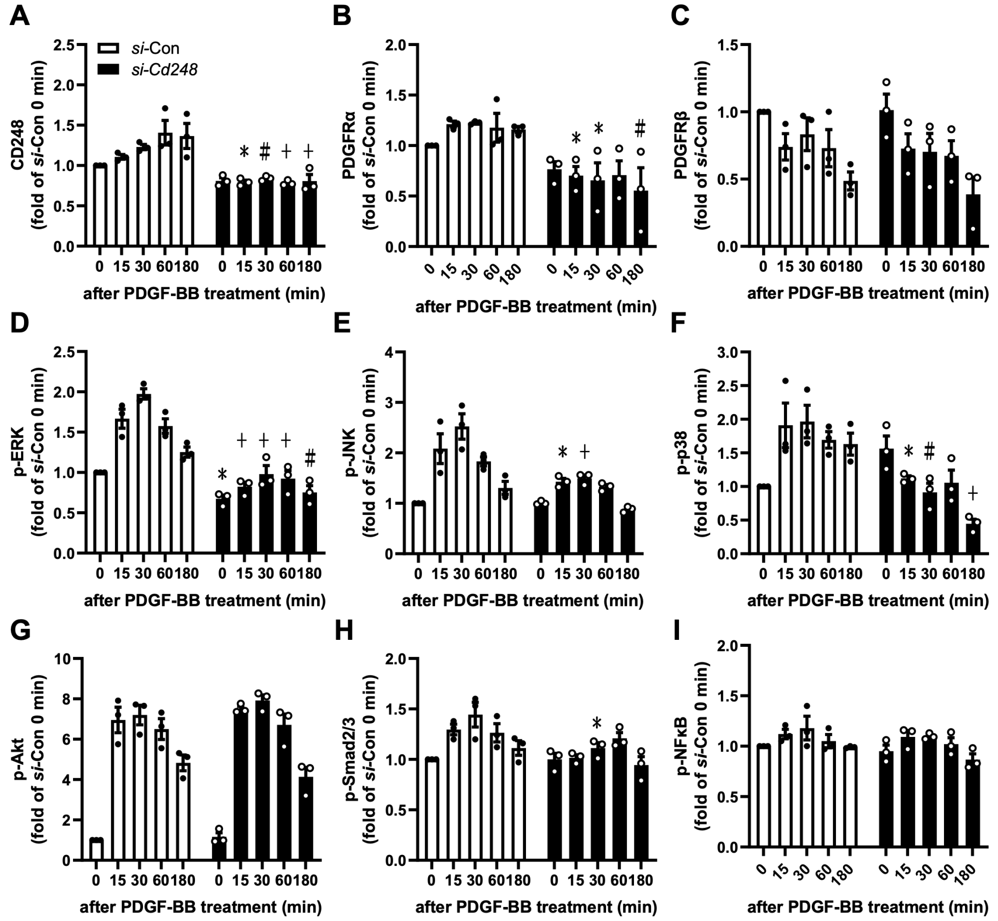

Figure S19

Quantification for western blot images in Figure 7A. A-I, Quantification for the immunoblots of the time course of phosphorylation of several signaling molecules in response to PDGF-BB (20 ng/mL) treatment in control (si-Con) and CD248-knockdown (si-*Cd248*) A7r5 cells as shown in Figure 7A (N=3). **P*<0.05, #*P*<0.01, and +*P*<0.001 by two-way ANOVA with Bonferroni correction.

Supplementary Figure 20

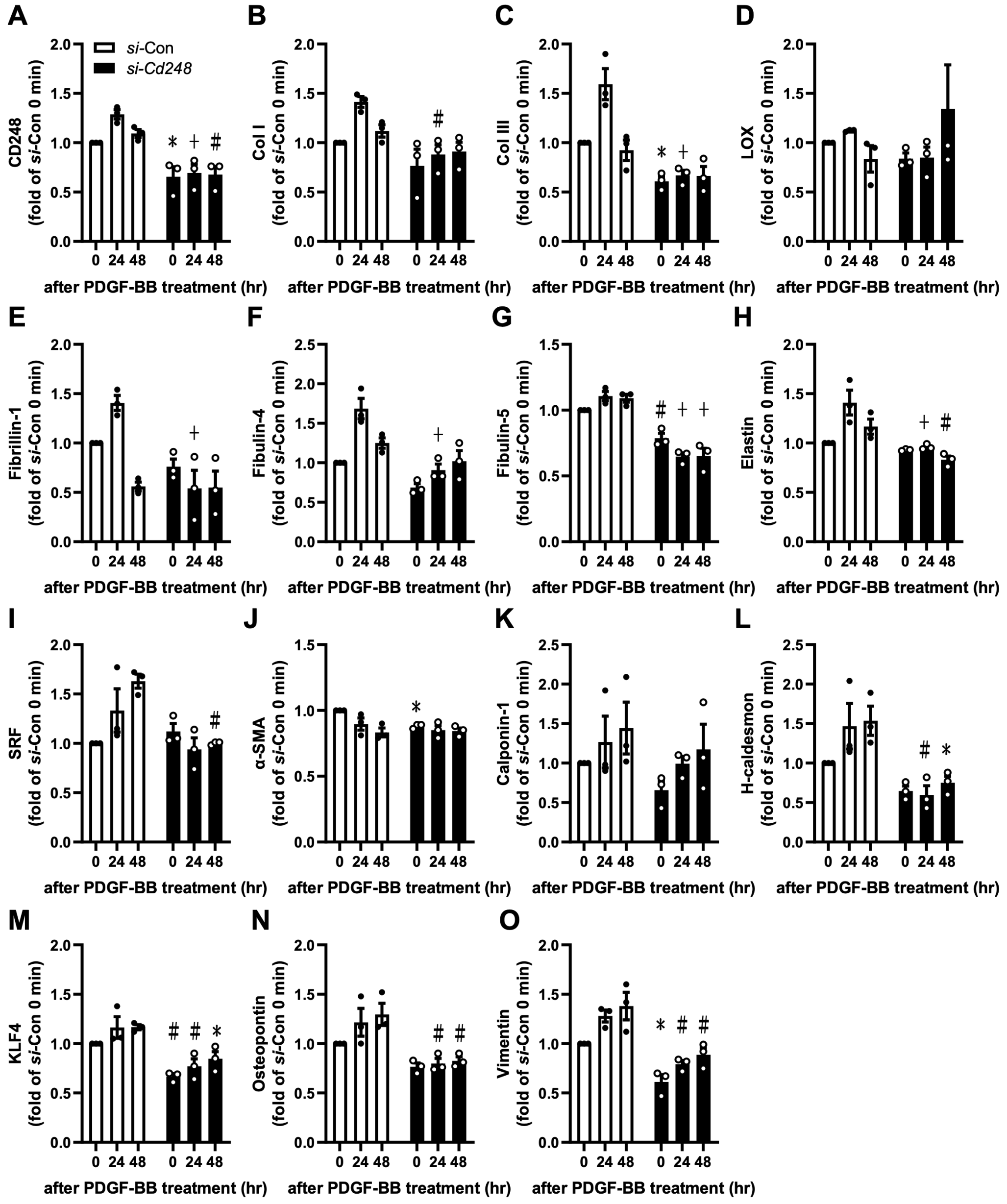

Figure S20

Quantification for western blot images in Figure 7B and 7C. A-H, Quantification for the immunoblots of the time course of protein levels of elastin and collagen fiber components in response to PDGF-BB treatment in CD248-knockdown A7r5 cells as shown in Figure 7B (N=3). **I-O,** Quantification for the immunoblots of VSMC-specific contractile and synthetic biomarkers as shown in Figure 7C (N=3). *P<0.05, $^{\#}P$<0.01, and ^{+}P<0.001 by two-way ANOVA with Bonferroni correction.

Supplementary Figure 21

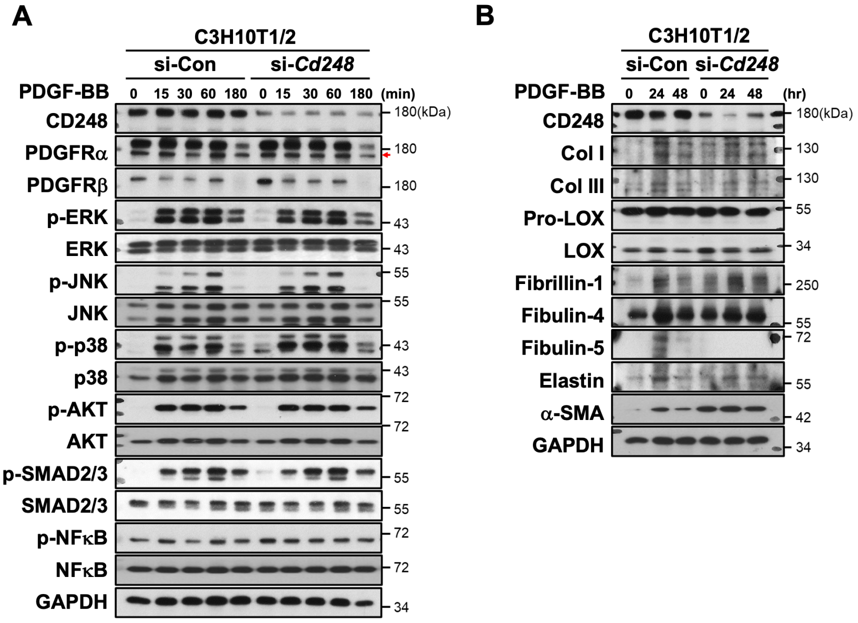

Figure S21

Normal PDGF-induced responses in CD248-knockdown fibroblasts. A, Time course of phosphorylation of several signaling molecules in response to PDGF-BB (20 ng/mL) treatment in control (si-Con) and CD248-knockdown (si-*Cd248*) C3H10T1/2 cells. **B,** Time course of protein levels of elastin and collagen fiber components and α-SMA in response to PDGF-BB treatment in CD248-knockdown C3H10T1/2 cells.

Supplementary Figure 21

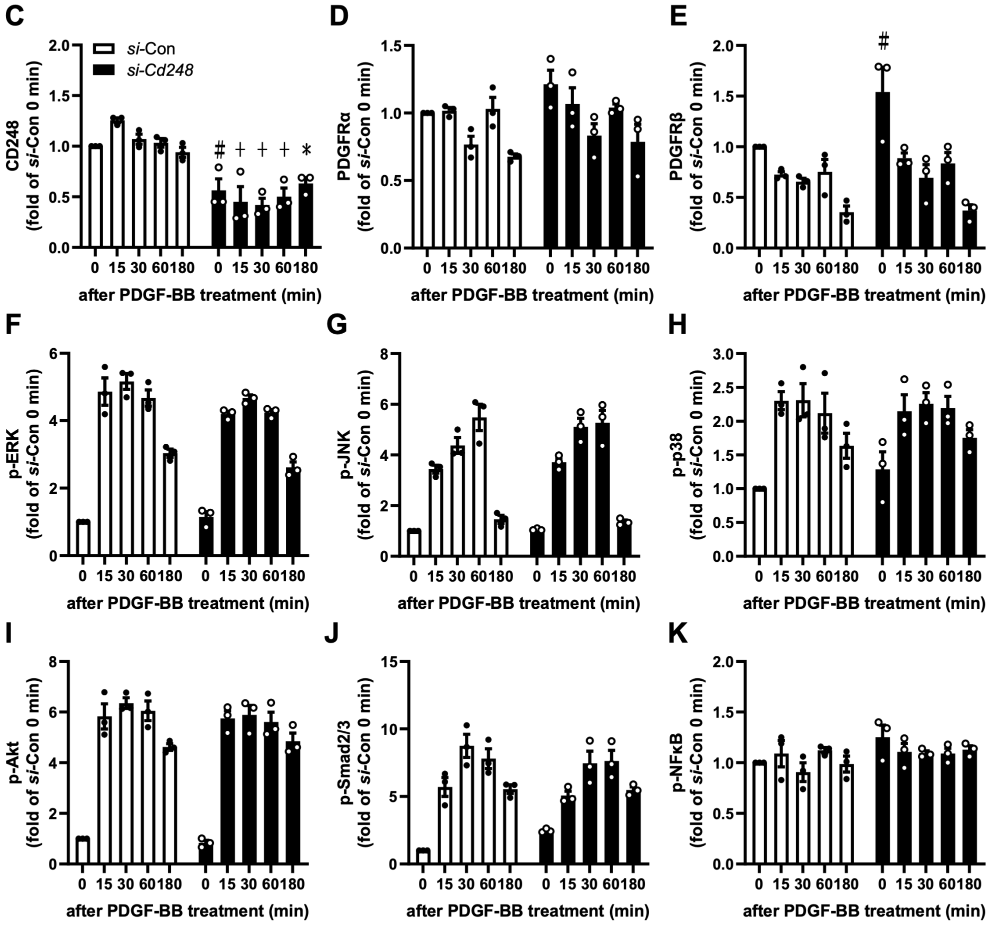

Figure S21 (Continued)

C-K, Quantification for the immunoblots of the time course of phosphorylation of several signaling molecules in response to PDGF-BB (20 ng/mL) treatment in control (si-Con) and CD248-knockdown (si-*Cd248*) C3H10T1/2 cells as shown in Figure S21A (N=3). **P*<0.05, #*P*<0.01, and +*P*<0.001 by two-way ANOVA with Bonferroni correction.

Supplementary Figure 21

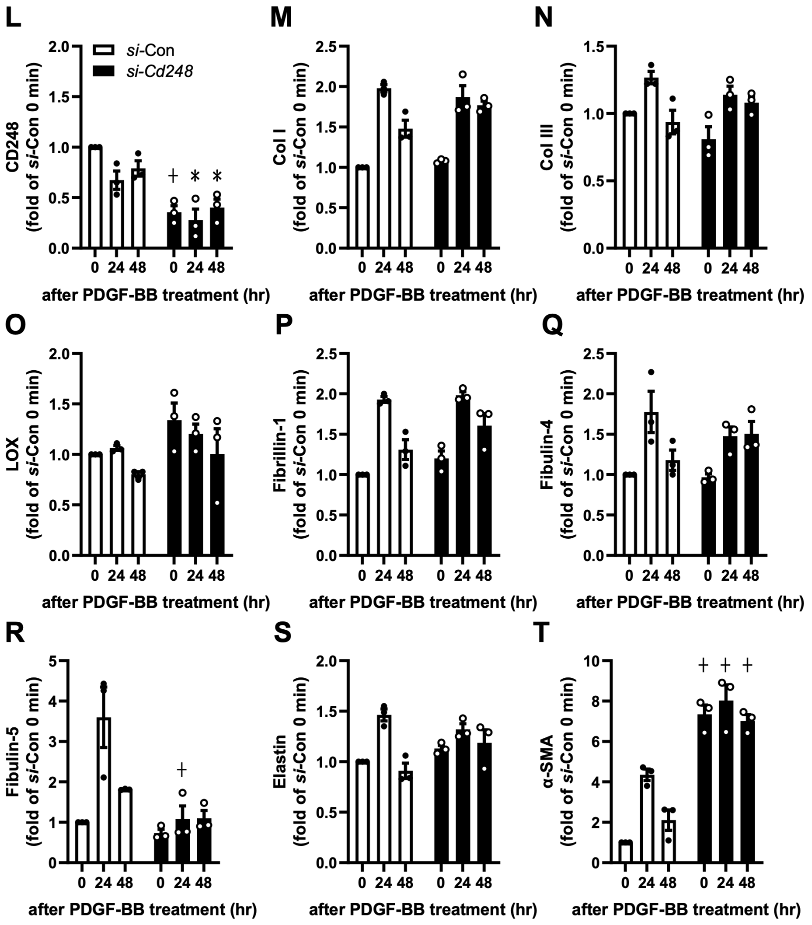

Figure S21 (Continued)

L-T, Quantification for the immunoblots of the time course of protein levels of elastin and collagen fiber components and α-SMA in response to PDGF-BB treatment in CD248-knockdown C3H10T1/2 cells as shown in Figure S21B (N=3). **P*<0.05 and +*P*<0.001 by two-way ANOVA with Bonferroni correction.

Supplementary Figure 22

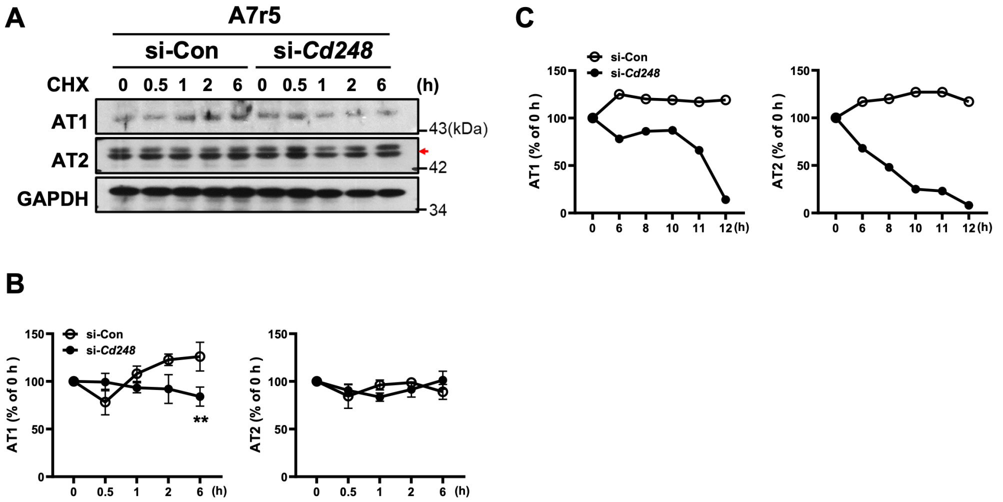

Figure S22

Increased Ang II and PDGF receptors protein stability in CD248-knockdown vascular smooth muscle cells (VSMCs). A, Representative immunoblot and quantification (**B**) of Ang II receptors (AT1 and AT2) after cycloheximide (CHX, 10 ug/mL) treatment at different time points (up to 6 hours) in control (si-Con) and CD248-knockdown (si-*Cd248*) A7r5 cells. The relative protein ratio to time 0 is calculated from three independent experiments. **C,** Quantification of Ang II receptors (AT1 and AT2) signal intensities after CHX treatment at different time points (up to 12 hours) in CD248-knockdown A7r5 cells as shown in Figure 8C (N=1). $^{**}P<0.01$ by two-way ANOVA with Bonferroni correction.

Supplementary Figure 23

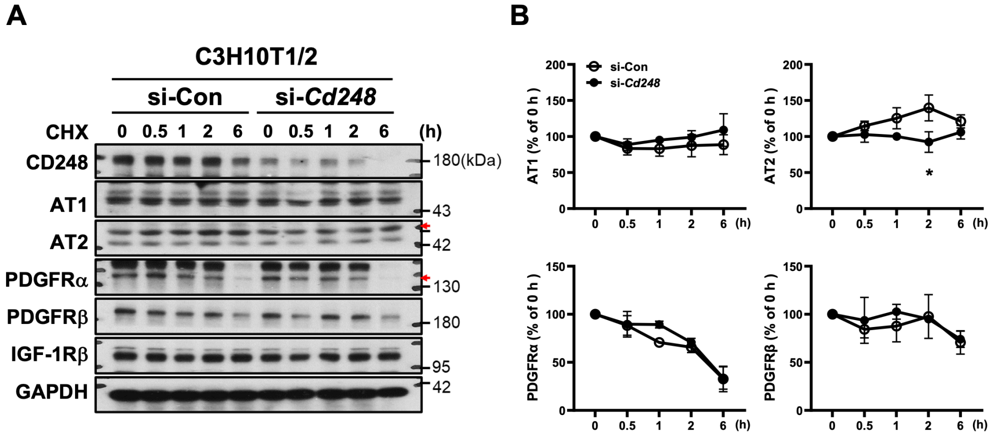

422 **Figure S23**

423 **Normal Ang II and PDGF receptors protein stability in CD248-knockdown**
424 **fibroblasts. A,** Representative immunoblot and quantification (**B**) of Ang II receptors
425 (AT1 and AT2), PDGF receptors (PDGFRα and PDGFRβ), and IGF-1Rβ after CHX
426 treatment at different time points in control (si-Con) and CD248-knockdown (si-*Cd248*)
427 C3H10T1/2 cells. The relative protein ratio to time 0 is calculated from three
428 independent experiments. **P*<0.05 by two-way ANOVA with Bonferroni correction.

Supplementary Figure 24

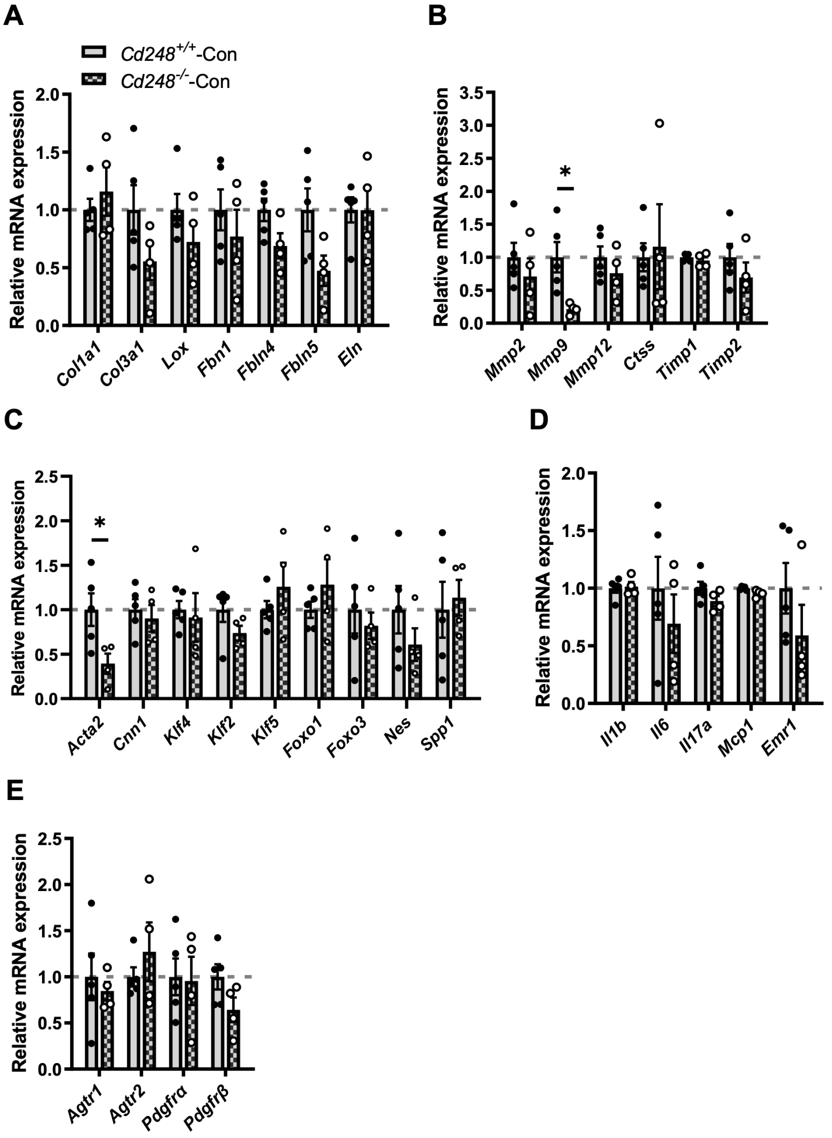

Figure S24

mRNA levels in the abdominal aorta (AA) from basal *Cd248*$^{+/+}$ and *Cd248*$^{-/-}$ mice.

A, mRNA levels of collagens and elastic fibers components and elastolytic enzymes (**B**), VSMC phenotypic markers and transcription factors (**C**), inflammatory cytokines and macrophage markers (**D**), and AT1 (*Agtr1*), AT2 (*Agtr2*), PDGFRα, and PDGFRβ (**E**) in control *Cd248*$^{-/-}$ and *Cd248*$^{+/+}$ aorta are shown relative to the mean level in control *Cd248*$^{+/+}$ aorta, which is set as 1.0 (N=4-5). *P<0.05 by Mann–Whitney *U* test.

Supplementary Figure 25

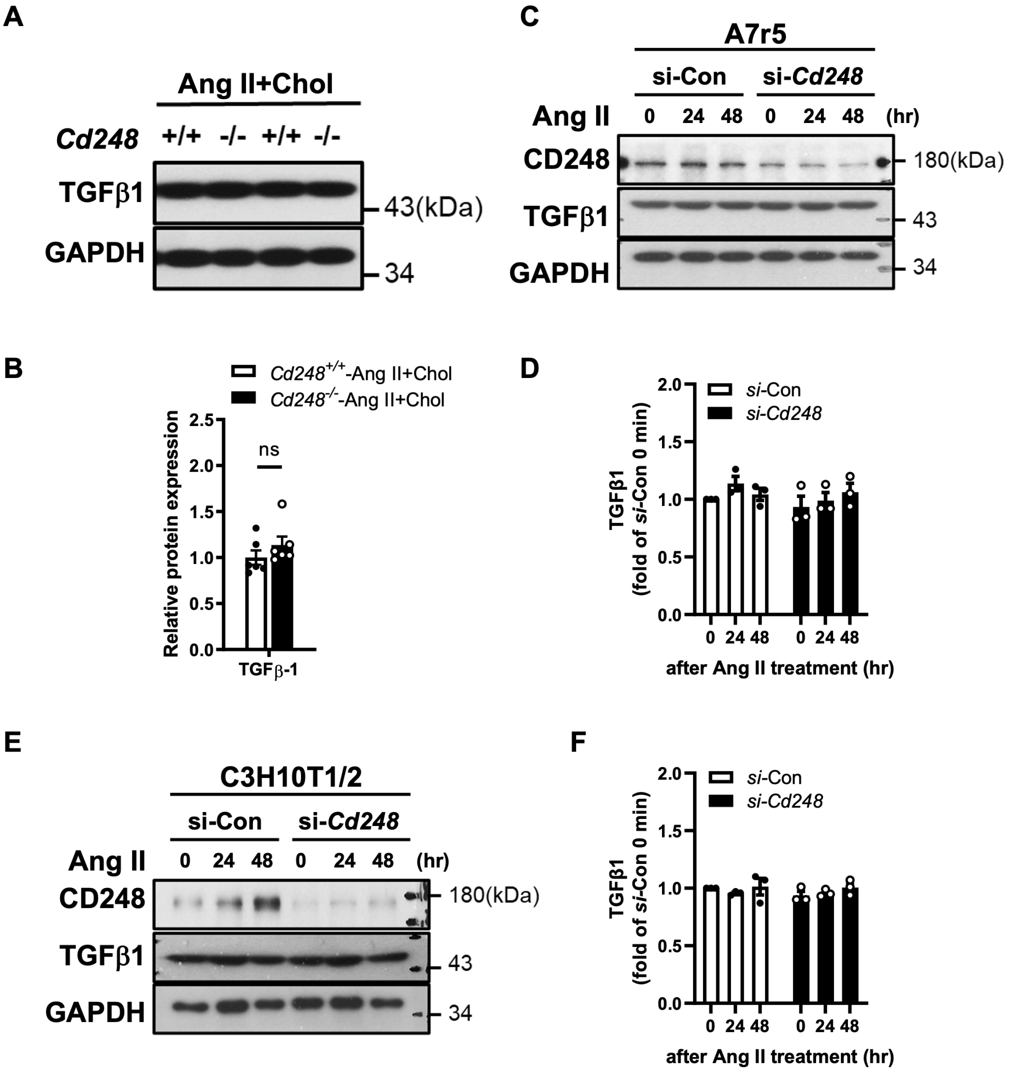

Figure S25

The levels of TGFβ-1 in *Cd248* deficiency after Ang II response. A, Representative immunoblot and quantification (**B**) of TGFβ-1 in *Cd248*$^{-/-}$ aorta after Ang II+Chol infusion (N=6). **C,** Time course and quantification (**D**) of protein levels of TGFβ-1 in response to Ang II (100 nM) treatment in control (si-Con) and CD248-knockdown (si-*Cd248*) A7r5 cells (N=3). **E,** Time course and quantification (**F**) of protein levels of TGFβ-1 in response to Ang II treatment in CD248-knockdown C3H10T1/2 cells (N=3). No significance by two-way ANOVA with Bonferroni correction.

Supplementary Figure 26

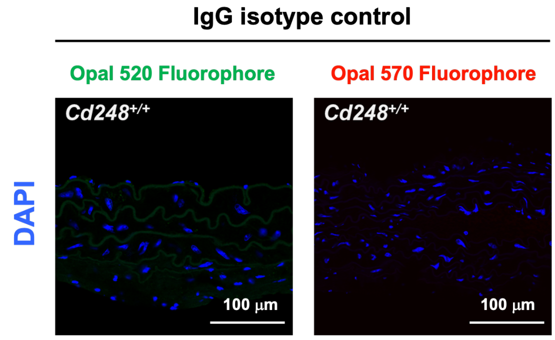

Figure S26

Isotype-matched control staining of abdominal aorta (AA) in Ang II+Chol *Cd248*$^{+/+}$ mice. Representative confocal images of normal mouse IgG using Polymer HRP-conjugated secondary antibodies with Opal 520 fluorophore (*green*) or Opal 570 fluorophore (*red*) in the AA of Ang II+Chol *Cd248*$^{+/+}$ mice. The results showed negative signal for the IgG isotype control. Scale bars are 100 µm.

Supplementary Tables

Table S1. The demographic characteristics of human subjects.

Cohort	Control	Aortic Aneurysm
**n**	3	8
**M : F**	3:0	6:2
**Age (y)# ± SD**	71.7 ± 4.5	65 ± 11.5
**Weight (kg) ± SD**	66.5 ± 5.9	63.7 ± 8.8
**Large AA (≥ 5 cm)**	0/3	8/8
**AAA**	0/3	3/8
**TAA**	0/3	3/8
**Root**	0/3	2/8
**Hypertension**	1/3	3/8
**Dyslipidemia**	0/3	2/8
**Heart failure**	2/3	2/8
**Paroxysmal atrial fibrillation**	0/3	1/8
**Intraluminal thrombus**	0/3	3/8

Values are mean ± SD.

#indicates the age of patients undergoing operation.

Table S2. The information for control individuals and aneurysm patients.

Group	Gender	Race	Age$^{\#}$	DIAGNOSIS/PROCEDURE	Figure
Con-1	M	Asian	76	1. Aortic stenosis without aortic dilation<br>2. Hypertension	Figure 1A
Con-2	M	Asian	72	Critical aortic stenosis with congestive heart failure without aortic dilation	Figure S1
Con-3	M	Asian	67	Severe aortic stenosis and congestive heart failure without aortic dilation	Figure S1
AAA-1	M	Asian	53	1. Abdominal aortic aneurysm<br>2. Coronary artery disease (3-V-D) s/p Left anterior descending artery percutaneous coronary intervention	Figure 1A
AAA-2	M	Asian	64	1. Infrarenal abdominal aortic aneurysm with bilateral iliac involvement<br>2. Right iliac artery to left iliac vein fistulae<br>3. High output heart failure<br>4. Acute on chronic renal failure<br>5. Intraluminal thrombus by computed tomography angiography (CTA)	Figure S1
AAA-3	M	Asian	63	1. Abdominal aortic aneurysm<br>2. Coronary artery disease (LM + 3-V-D) s/p coronary artery bypass graft *4 with partial venous graft failure<br>3. Hypertension<br>4. Dyslipidemia<br>5. Paroxysmal atrial fibrillation<br>6. Intraluminal thrombus by CTA	Figure S1
TAA-1	F	Asian	61	1. Sinus valsalva aneurysm (at least 8 cm in diameter) with thrombosis formation (NCC) (at least 2/3 aneurysm space)<br>2. Severe aortic regurgitation with congestive heart failure<br>3. Ascending aortic aneurysm (5.0 cm in diameter)<br>4. Hypertension<br>5. Intraluminal thrombus by CTA	Figure S2
TAA-2	M	Asian	n/a	1. Ascending aortic aneurysm<br>2. Aortic root aneurysm with severe aortic regurgitation<br>3. Hypertension	Figure S2

TAA-3	F	Asian	84	1. Ascending aortic aneurysm with arch involvement<br>2. Moderate aortic regurgitation<br>3. Dyslipidemia	Figure S2
Root-1	M	Asian	n/a	1. Coronary artery disease (LM+2-V-D)<br>2. Aortic root aneurysm with moderate aortic regurgitation	Figure S2
Root-2	M	Asian	n/a	Aortic root aneurysm with severe aortic regurgitation and bicuspid aortic valve	Figure S2

#indicates the age of patients undergoing operation.

Table S3. List of antibodies used in experiments.

Antigen	Host	Cat.	Source	Applications
**Primary antibodies**				
CD248	Rabbit<br>Mouse	18160-1-AP,<br>60170-1-Ig	Proteintech	WB, IHC
CD248	Rabbit	HPA051856	ATLAS	WB, IHC, IF
phospho-ERK	Mouse	sc-7383	Santa Cruz	WB
ERK	Rabbit	sc-94	Santa Cruz	WB
phospho-JNK	Rabbit	#4668	Cell Signaling	WB
JNK	Rabbit	#9252	Cell Signaling	WB
phospho-p38	Rabbit	#9211	Cell Signaling	WB, IF
p38	Rabbit	#9212	Cell Signaling	WB
phospho-AKT	Rabbit	#4058	Cell Signaling	WB
AKT	Rabbit	#9272	Cell Signaling	WB
phospho-SMAD2/3	Rabbit	ab52903	Abcam	WB
SMAD2/3	Mouse	MABT521	Millipore	WB
phospho-NF-*k*B	Rabbit	MA5-15160	Invitrogen	WB
NF-*k*B	Rabbit	sc-372	Santa Cruz	WB
AT1	Rabbit	DF4910	Affinity<br>Biosciences	WB, IF
AT2	Mouse	ab254561	Abcam	WB, IF
PDGFRα	Rabbit	bsm-52829R	Bioss	WB, IF
PDGFRβ	Mouse	sc-374573	Santa Cruz	WB, IF
phospho-PDGFRβ	Rabbit	GTX133525	GeneTex	WB
IGF-1Rβ	Rabbit	#3027	Cell Signaling	WB
TGFβR-II	Rabbit	bs-0117R	Bioss	WB
Collagen I	Rabbit	ab34710	Abcam	WB, IF
Collagen III	Rabbit	ab7778	Abcam	WB
Pro-LOX	Rabbit	ab174316	Abcam	WB
LOX	Rabbit	ab31238	Abcam	WB
Fibrillin-1	Rabbit	ab53076	Abcam	WB
Fibulin-4	Rabbit	3647-S-EX	Epitomics	WB
Fibulin-5	Rabbit	12188-1-AP	Proteintech	WB
Elastin	Rabbit	CL55041-AP<br>MAB-2503	Cedarlane Labs	WB
SRF	Mouse	sc-25290	Santa Cruz	WB
α-SMA	Mouse	A5228	Sigma-Aldrich	WB, IHC, IF
Calponin-1	Rabbit	ab46794	Abcam	WB
H-caldesmon	Mouse	sc-58703	Santa Cruz	WB
KLF4	Mouse	sc-393462	Santa Cruz	WB
Osteopontin	Rabbit	GTX28448	GeneTex	WB
Vimentin	Rabbit	ab92547	Abcam	WB, IHC, IF
F4/80	Rabbit	#70076	Cell Signaling	IHC
vWF	Rabbit	AB7356	Merck Millipore	IF

IgG	Mouse	500-M00	Peprotech	IHC, IF
**Secondary antibodies**				
anti-Mouse IgG-HRP	Goat	20102	Leadgene	WB
anti-Rabbit IgG-HRP	Goat	20202	Leadgene	WB

*WB, western blotting; IHC, Immunohistochemistry; IF, Immunofluorescence

Table S4. Sequences of primers used for real-time PCR.

Gene		Sequence
mu/rat *Cd248*	Forward	ACA GGA TGG AGA GCC TCA GA
	Reverse	AGG TAG TGT GGG TCG AGT GG
mu *Col1a1*	Forward	TCA GAG GCG AAG GCA ACA GTC
	Reverse	GCA GGC GGG AGG TCT TGG
mu *Col3a1*	Forward	GAC AGA TTC TGG TGC AGA GA
	Reverse	CAT CAA CGA CAT CTT CAG GAA T
mu *Lox*	Forward	GTC ACC AAC ATT ACC ACA GCA
	Reverse	CAT AAC ATC CAG GAC TCA ATC C
mu *Fbn1*	Forward	TCA TCG GAG GCT ATA GGT GTA GCT
	Reverse	CAC TCA GGC ACT CGT TTT CAT C
mu *Fbln4*	Forward	GGG TTA TTT GTG TCT GCC TCG
	Reverse	TGG TAG GAG CCA GGA AGG TT
mu *Fbln5*	Forward	TCC AAC TAC CCC ACG ATT TCA AG
	Reverse	GGC AGT AAC CAT AGC GAC ATT C
mu *Eln*	Forward	CTG CCA AAG CTG CCA AAT AC
	Reverse	CTC CAG CTC CAA CAC CAT AG
mu *Mmp2*	Forward	CCC CGA TGC TGA TAC TGA
	Reverse	CTG TCC GCC AAA TAA ACC
rat *Mmp2*	Forward	ATC GCA GAC TCC TGG AAT GC
	Reverse	TCA GGT AAT AAG CAC CCT TGA AGA
mu *Mmp9*	Forward	CCT GGA ACT CAC ACG ACA TCT TC
	Reverse	TGG AAA CTC ACA CGC CAG AA
rat *Mmp9*	Forward	TGG ATC CCC AGA GCG TTAC T
	Reverse	AAT AGG CCT TGT CTT GGT AGT GAAA
mu *Mmp12*	Forward	CCC ACT TCG CCA AAA GGT TT
	Reverse	CAT GAG CTC CTG CCT CAC ATC
mu *Ctss*	Forward	GGTTGGCTATGGGACTCTTG
	Reverse	GCA ATT CCG CAG TGA TTT TT
mu *Timp1*	Forward	ATT CAA GGC TGT GGG AAA TG
	Reverse	CTC AGA GTA CGC CAG GGA AC
mu *Timp2*	Forward	CAC GCT TAG CAT CAC CCA
	Reverse	TGA CCC AGT CCA TCC AGA G
mu *Acta2*	Forward	CGG CTT CGC TGG TGA TGA TG
	Reverse	TCC CTC TCT TGC TCT GGG CTT
mu *Cnn1*	Forward	ACC AAC CAT ACA CAA GTT CAG TCC
	Reverse	CCA ATG ATG TTC CTG CCT TCT CTC
mu *Klf4*	Forward	CCC CTC TCT CCA TTA TCA AG
	Reverse	CTC TTG GTA TAG GTT TTG CC
mu *Klf2*	Forward	AGC CTA TCT TGC CGT CCT T
	Reverse	CCA ACA CGT TGT TTA GGT CCT C
mu *Klf5*	Forward	ACC ATT TTC AGC CAC CAG AG
	Reverse	GTC TGG TGG GAG CTG AAG A
mu *Foxo1*	Forward	GTA CGC CGA CCT CAT CAC CA
	Reverse	TGC TGT CGC CCT TAT CCT TG
mu *Foxo3*	Forward	AGT GGA TGG TGC GCT GTG T
	Reverse	CTG TGC AGG GAC AGG TTG T

mu *Nes*	Forward	CAG CAA CTG GCA CAC CTC AA
	Reverse	CTG GTA TCC CAA GGA AAT GCA
mu *Spp1*	Forward	GGA TGA ATC TGA CGA ATC TC
	Reverse	GCA TCA GGA TAC TGT TCA TC
mu *Il1b*	Forward	CAA CCA ACA AGT GAT ATT CTC CAT G
	Reverse	GAT CCA CAC TCT CCA GCT GCA
mu *Il6*	Forward	AGG ATA CCA CTC CCA ACA GAC
	Reverse	GTG CAT CAT CGT TGT TCA TAC
mu *Il17a*	Forward	ACG TTT CTC AGC AAA CTT AC
	Reverse	CCC CTT TAC ACC TTC TTT TC
mu *Mcp1*	Forward	GGC TCA GCC AGA TGC AGT TAA
	Reverse	AGC AAG ATG ATC CCA ATG AGT AGG
mu *Emr1*	Forward	CTT TGG CTA TGG GCT TCC AGT C
	Reverse	GCA AGG AGG ACA GAG TTT ATC GTG
mu *Vwf*	Forward	CCT GTC TAT CAA TGA AGT GTG
	Reverse	TTC TGC AGA TAC AAG TGT TG
mu *Cd31*	Forward	TCA GAA CCC ATC AGG AGT GAA TAC G
	Reverse	TGC TTG GAG GTG GCT ACA ATC G
mu *Dcn*	Forward	TAA GTA TAT CCA GGT CGT CTA C
	Reverse	CAC ATA GAC ACA TCT GAA GG
mu *Fsp1*	Forward	CCT CTT TGC CTG AGT ATT TG
	Reverse	TAT TCA GCA CTT CCT CTC TC
mu *Agtr1*	Forward	GCT ATA AAT ATG GAA GTG CCT C
	Reverse	TCA TAT GTT AAG TCC GGG AG
rat *Agtr1*	Forward	TCC CTG AGT TAA CAT ATG AGA G
	Reverse	TGT TTT TCT GGG TTG AGT TG
mu/rat *Agtr2*	Forward	AAA AGG TGT CCA GCA TTT AC
	Reverse	AAA AGA ACC AAA CAC TTT GC
mu *Pdgfrα*	Forward	CTA GTT CCT GCA ACC ATT TTG
	Reverse	ATA TTT GAG ACA TTG CTG GC
rat *Pdgfrα*	Forward	GAG ATT ATG AAT GTG CTG CC
	Reverse	TTT CTC GTG AAC AGA AAT GG
mu *Pdgfrβ*	Forward	ATC TCT GTG ATC GAG AAT GG
	Reverse	AGT AGA CAA AAC TAA CTC GC
rat *Pdgfrβ*	Forward	CAT TGG CTC CAT TCT TCA TAT C
	Reverse	ACA TAG CCA TTT TCG ATC AC
mu *Gapdh*	Forward	TGA CGT GCC GCC TGG AGA AA
	Reverse	AGT GTA GCC CAA GAT GCC CTT
mu/rat *Ubc*	Forward	TGA CAA TGC AGA TCT TTG TG
	Reverse	ACT CCT TCT GGA TGT TGT AG
mu/rat *Actb*	Forward	ACT GCC GCA TCC TCT TCC TC
	Reverse	TGC CAC AGG ATT CCA TAC CC
